# The economic ramifications of liver cancer on a global, regional, and national scale

**DOI:** 10.3389/fmed.2025.1694588

**Published:** 2025-11-03

**Authors:** Jie Yu, Qiaoyi Chen, Jin Ma

**Affiliations:** ^1^Department of Radiology, Yongchuan People’s Hospital of Chongqing, Chongqing, China; ^2^Department of Radiology, Chongqing Traditional Chinese Medicine Hospital, Chongqing, China

**Keywords:** liver cancer, macroeconomic burden, value of lost welfare (VLW), disability-adjusted life years (DALY), economic impact

## Abstract

**Background:**

A comprehension of the macroeconomic losses on a worldwide, regional, and national scale attributable to liver cancer is crucial for the optimal distribution of medical and research materials. The authors conducted an investigation into the macroeconomic impacts of the strain imposed by liver cancer in 2021 across 185 nations.

**Methods:**

The data pertaining to disability-adjusted life years (DALY) for liver cancer and its associated risk factors were sourced from the 2021 records of the Global Burden of Disease investigation. Information pertaining to GDP, modified for purchasing power parity (PPP), originated from the World Bank; the integration of GDP and DALY data facilitated the estimation of macroeconomic losses through the application of a value of lost welfare (VLW) methodology. Every finding is articulated in 2021 international US dollars, calibrated for PPP.

**Outcomes:**

In the year 2021, the VLW resulting from liver cancer worldwide amounted to $141.95 B, representing 0.15% of the worldwide GDP. The worldwide VLW/GDP ratio for alcohol-related liver cancer was 0.033% (VLW = $31.835 B) Hepatitis B-associated liver cancer prevalence was 0.041% (VLW = $38.667 B) Hepatitis C-associated liver cancer prevalence was 0.056% (VLW = $53.268 B) incidence of NASH-related liver cancer was 0.012% (VLW = $11.653 B) the incidence of liver cancer attributed to alternative factors was recorded at 0.007% (VLW = $6.728 B). The East Asia, Southeast Asia, and Oceania super-region recorded the greatest VLW/GDP for liver cancer overall was 0.19%, with VLW of $39.08 B, the high-income super-region accounted for the second (VLW/GDP = 0.16%; VLW = $88.00 B).

**Conclusion:**

The global macroeconomic burden attributable to liver cancer is substantial, with far-reaching implications for productivity losses and healthcare expenditure. These evidence-based economic estimates provide a compelling rationale for strategic resource allocation towards liver cancer control programs.

## Introduction

Liver cancer represents a neoplastic growth that manifests within the hepatic tissue (1–3). It stands as the third leading cause of death attributed to cancerous growths and remains a considerable challenge for global medical systems. In almost 50% of nations, the prevalence of liver cancer is on the rise. According to statistics, in the year 2020, the global incidence of liver cancer reached approximately 900,000 new incidents, and the death rate due to liver cancer in the same year is as high as 830,000 (4, 5). Due to the hidden early symptoms of liver cancer, majority of individuals are diagnosed during the middle to late stages of their condition, which leads to high treatment cost and poor prognosis, and imposes significant health and economic challenges on people, their families, and the community at large (6). Moreover, the elevated occurrence of liver cancer is intricately linked to chronic HBV, HCV, alcoholic liver disease, and NAFLD. The prevalence trends of these etiological factors exhibit notable variations throughout various nations and territories, thereby intensifying the intricacies associated with the strategies for preventing and managing liver cancer (2, 7, 8). In this context, measuring the weight of illness of liver cancer and its implications on the broader economy are critical.

The Global Burden of Disease Study system evaluates trends within the epidemiology of liver cancer and its effects on worldwide health (9). Previous studies show that although HBV vaccination has significantly reduced liver cancer incidence in some countries (10, 11), nonetheless, the impact of liver cancer resulting from exposure to HCV, metabolic liver disease, and alcohol abuse continues to rise (1, 12).

Previous large-scale studies on the economic burden of liver cancer have typically employed cost-of-illness or human capital approaches within single countries or regions. For example, Cao et al. (13) estimated the population-level economic burden of liver cancer in China from 2019 to 2030 using a prevalence-based approach covering direct and indirect costs but without applying welfare-based metrics. Similarly, a recent cross-sectional cost-of-illness study in China assessed patient-level expenditures across treatment phases for major cancers, including liver cancer, focusing largely on direct medical expenditures and productivity losses (14). Global cancer cost projections such as those published in JAMA Oncology have estimated the macroeconomic cost of multiple cancer types (including liver cancer) across more than 200 countries, yet did not disaggregate by etiological subtypes or employ welfare-based approaches sensitive to income elasticity (15). To our knowledge, systematic application of the value-of-a-statistical-life-year (VLW) framework to liver cancer on a global scale—at super-regional and national levels with etiological disaggregation and sensitivity analyses—remains very limited. By combining VLW estimates with GBD-derived DALYs, our study complements and extends the existing literature, providing a welfare-based perspective on the economic burden of liver cancer that goes beyond direct costs or productivity losses alone.

The VLW serves as a progressively advanced macroeconomic assessment tool that quantifies the economic costs of current diseases to social welfare through standardized methods (16, 17). The VLW model is grounded in the value of statistical life (VSL) and DALYs, and is standardized in conjunction with the national economic level (18). Broadly speaking, VSL measures the financial amount that a person is prepared to spend to lower a specific risk of death, while VLW extends this theory to construct a multi-dimensional evaluation system: it includes both direct market economic losses (such as quantifiable measures of productivity loss and health care expenditures) and systematic inclusion of losses in welfare that are not captured by market transactions, encompassing the intrinsic value of health, quality of life, and various social dimensions that resist quantification in monetary terms (18, 19). Compared with traditional cost–benefit analysis, VLW method builds a more comprehensive framework for socio-economic impact assessment (20, 21). It is based on this advantage that the WHO has advocated for the adoption of payment willingness methodologies, including VLW when developing macroeconomic models of health policy (22).

In this scenario, the researchers quantify 2021 macroeconomic losses from liver cancer and its causes using GBD study DALY data from 185 high, middle, and low income nations.

## Methods

### Sources of data

The data on DALYs for liver cancer was obtained from the GBD 2021 records (23). Liver cancer, as outlined in the GBD investigation encompasses ICD-10 codes C22.0–22.4 and 22.7–22.9 (C22.0 = Hepatocellular carcinoma, C22.1 = Intrahepatic bile duct carcinoma, C22.2 = Hepatoblastoma, C22.3 = Angiosarcoma of liver, C22.4 = Other sarcomas of liver, C22.7 = Other specified liver cancers, C22.8 = Overlapping lesion of liver, C22.9 = Liver cancer, unspecified). Age-stratified DALY rates were gathered for 185 countries encompassing all available age categories (23). Country-level GDP data were derived from the World Bank’s World Development Indicators records. All economic values were standardized to 2021 USD utilizing PPP conversion factors to ensure cross-national comparability (24). Consistent with established GBD methodology, we categorized countries into predefined super-regions for comparative analysis (23). Seven super-regions were identified in the GBD investigation: (1) Central Asia, Eastern Europe, and Central Europe; (2) High-income; (3) The Caribbean and Latin America; (4) The Middle East and North Africa; (5) South Asia; (6) Oceania, East Asia, and Southeast Asia; (7) Sub-Saharan Africa (23).

### Assessment of VLW

VLW model quantifies welfare losses due to illness based on VSL, that includes nonmarket losses, like missed leisure time, nonhealth-related consumption, and the intrinsic value of being healthy (18). The VSL quantifies the highest monetary sum that a person is prepared to spend in order to decrease the likelihood of mortality. This measure quantifies the economic value of the risk of death, and when integrated with DALYs, VSL is able to assess the overall the broader economic impacts of a specific illness (16, 25). Given that VSL benchmark data mainly originates from empirical studies conducted in wealthy nations, it is essential to utilize established VSL projections from the United States Department of Transportation (USDOT) to achieve a standardized estimation for all nations. Therefore, this study is derived using the following standardized calculation formula (25, 26):


$${\mathrm{VSL}}_{\text{peak},i}={\mathrm{VSL}}_{\text{peak},\mathrm{USA}}\times {\left({\mathrm{GDP}}_{i}/{\mathrm{GDP}}_{\mathrm{USA}}\right)}^{\mathrm{IE}}$$

Following the adjustment for PPP, the GDP of a particular nation is transformed into comparable data that aligns with the United States benchmark (27). The approach for passing VSL projections across countries relies on the income elasticity (IE) parameter of VSL, with the IE-VSL recognized as a gold standard of 0.55 for transfers among high-income nations. However, recent research indicates that more conservative elasticity coefficients ranging from 1.0 to 1.5 are more suitable when drawing conclusions from high-income to low-income country contexts (17, 28). To ensure conservative estimation, our base case applies a unity income elasticity (IE = 1.0). In addition, to assess the robustness of our results to the income elasticity assumption, we recalculated VLW and VLW/GDP using IE values of 0.55 and 1.5, which represent the lower and upper bounds of the range reported in the literature. VSL_peak_ represents the age node of optimal willingness-to-pay (WTP) within an economy. Empirical analyses demonstrate this peak value predominantly occurs during middle age (95% CI, 45–55 years), a critical finding that provides the age-weighting parameter for calculating VSLY (18). In calculating the VSLY, the age-adjusted function *f*(*a*) was used to calibrate VSLpeak in this study. *f*(*a*) is a quadratic function, the specific age is characterized by parameter a, which illustrate the WTP of an individual across various life stages and that modifies a nation’s maximum VSL to reflect the VSLa according to the fraction of life already experienced (18). The final VLW was calculated by multiplying age-specific VSLYs with corresponding DALYs for each age group, followed by summation across all age cohorts. All financial evaluations are articulated in 2021 USD, modified for PPP (18). The VLW/GDP ratio serves as an indicator of the relative economic burden a disease places on a nation’s economy. By accounting for differences in national GDP, it enables more equitable comparisons across countries and regions (29).

This study utilized RStudio statistical software (RStudio PBC, Boston, MA) and strictly conformed with the Consolidated Health Economic Evaluation Reporting Standards standards (30).

## Results

**Figure 1:**
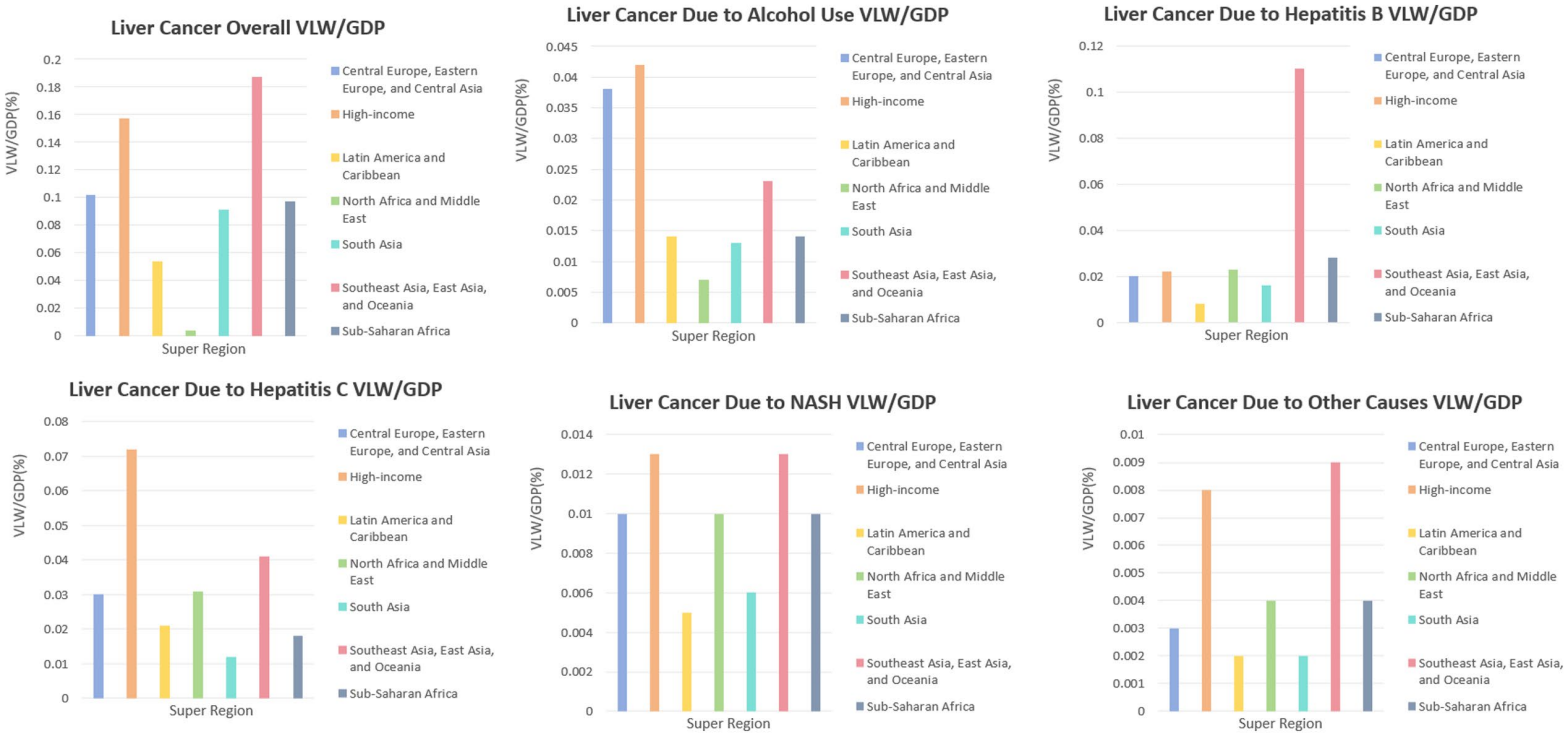
VLW/GDP in 2021 by GBD super-region for liver cancer and its pathogenic factors. VLW, value of lost welfare; GDP, gross domestic product.

In the year 2021, the VLW due to liver cancer on a global scale amounted to $141.95 B, representing 0.15% of the total worldwide GDP. The VLW as a proportion of GDP attributable to liver cancer was most evident in the East Asia, Southeast Asia, and Oceania super-region, with a VLW/GDP ratio of 0.19% and a total VLW of $39.08 B. The high-income super-region followed closely, exhibiting a VLW/GDP ratio of 0.16% and a VLW amounting to $88.00 B. Liver cancer exhibited a VLW/GDP of 0.10% in the Central Europe, Eastern Europe, and Central Asia super-region (VLW = $4.04 B); 0.097% in the Sub-Saharan Africa super-region (VLW = $1.806 B); 0.091% in the South Asia super-region (VLW = $3.626 B); 0.074% in the North Africa and Middle East super-region (VLW = $3.020 B); and 0.054% in the Latin America and Caribbean super-region (VLW = $2.329 B) (Figure 1).

The worldwide VLW attributable to liver cancer resulting from alcohol consumption in 2021 amounted to $31.835 B, representing 0.033% of the worldwide GDP. VLW as a percentage of GDP attributable to liver cancer from alcohol consumption was largest in the high-income super-region (VLW/GDP = 0.042%; VLW = $23.832). Liver cancer linked to drinking was 0.038% of VLW/GDP in the Central Asia and Central & Eastern Europe super-region (VLW = $1.512 B); 0.023% in the Southeast Asia, East Asia, and Oceania super-region (VLW = $4.826 B); 0.014% in the Latin America and Caribbean super-region (VLW = $0.611 B); 0.014% in the Sub-Saharan Africa super-region (VLW = $0.262 B); 0.013% in the South Asia super-region (VLW = $0.499 B); and 0.007% in the North Africa and Middle East super-region (VLW = $0.293 B) (Figure 1).

The worldwide VLW of liver cancer attributable to hepatitis B in 2021 was $38.667 B, representing 0.041% of the worldwide GDP. The value of liver cancer attributable to hepatitis B as a percentage of GDP was most significant in the Southeast Asia, East Asia, and Oceania super-region (VLW/GDP = 0.110%; VLW = $22.945). Liver cancer attributed to hepatitis B exhibited a VLW/GDP of 0.028% in the Sub-Saharan Africa super-region (VLW = $0.511 B); 0.023% in the North Africa and Middle East super-region (VLW = $0.934 B); 0.022% in the High-income super-region (VLW = $12.472 B); 0.020% in the Central Europe, Eastern Europe, and Central Asia super-region (VLW = $0.796 B); 0.016% in the South Asia super-region (VLW = $0.653 B); and 0.008% in the Latin America and Caribbean super-region (VLW = $0.356 B) (Figure 1).

In 2021, the worldwide value of VLW attributable to liver cancer resulting from hepatitis C was quantified at $53.268 B, representing 0.056% of the worldwide GDP. The value of lost work (VLW) as a proportion of GDP attributable to liver cancer resulting from hepatitis C was most pronounced in the High-income super-region, with a VLW/GDP ratio of 0.072% and a total VLW amounting to $40.545 B. The incidence of liver cancer attributable to hepatitis C exhibited a VLW/GDP of 0.041% within the Southeast Asia, East Asia, and Oceania super-region, corresponding to a VLW of $8.590 B. In contrast, the North Africa and Middle East super-region recorded a VLW/GDP of 0.031%, with a VLW of $1.245 B. The Central Europe, Eastern Europe, and Central Asia super-region reflected a VLW/GDP of 0.030%, amounting to a VLW of $1.185 B. The Latin America and Caribbean super-region presented a VLW/GDP of 0.021%, equating to a VLW of $0.893 B. Sub-Saharan Africa demonstrated a VLW/GDP of 0.018%, with a VLW of $0.329 B, while the South Asia super-region had a VLW/GDP of 0.012%, resulting in a VLW of $0.481 B (Figure 1).

The worldwide VLW of liver cancer attributable to NASH in 2021 amounted to $11.653 B, representing 0.012% of the worldwide GDP. The value of lost work (VLW) attributable to liver cancer resulting from NASH represented the most significant proportion of GDP within the High-income super-region, with a VLW/GDP ratio of 0.013% and a total VLW amounting to $7.548 B. The incidence of liver cancer attributed to NASH exhibited a VLW/GDP of 0.013% within the Southeast Asia, East Asia, and Oceania super-region, corresponding to a VLW of $2.643 B. In the North Africa and Middle East super-region, the figure was 0.010% with a VLW of $0.417 B. Similarly, the Central Europe, Eastern Europe, and Central Asia super-region reported a VLW/GDP of 0.010%, equating to a VLW of $0.386 B. The Sub-Saharan Africa super-region also recorded a 0.010% incidence, with a VLW of $0.177 B. In South Asia, the VLW/GDP was 0.006%, amounting to a VLW of $0.254 B, while the Latin America and Caribbean super-region had a VLW/GDP of 0.005% and a VLW of $0.228 B (Figure 1).

Global VLW of liver cancer due to other causes in 2021 was $6.728 B or 0.007% of the global GDP. The VLW as a proportion of GDP attributable to liver cancer from other factors was most pronounced in the Southeast Asia, East Asia, and Oceania super-region, with a VLW/GDP ratio of 0.009% and a total VLW amounting to $1.831 B. The incidence of liver cancer attributed to various causes exhibited a VLW/GDP of 0.008% within the High-income super-region (VLW = $4.383 B); 0.004% in the North Africa and Middle East super-region (VLW = $0.169 B); 0.004% in the Sub-Saharan Africa super-region (VLW = $0.067 B); 0.003% in the Central Europe, Eastern Europe, and Central Asia super-region (VLW = $0.115 B); 0.002% in the Latin America and Caribbean super-region (VLW = $0.103 B); and 0.002% in the South Asia super-region (VLW = $0.060 B) (Figure 1).

**Figure 2:**
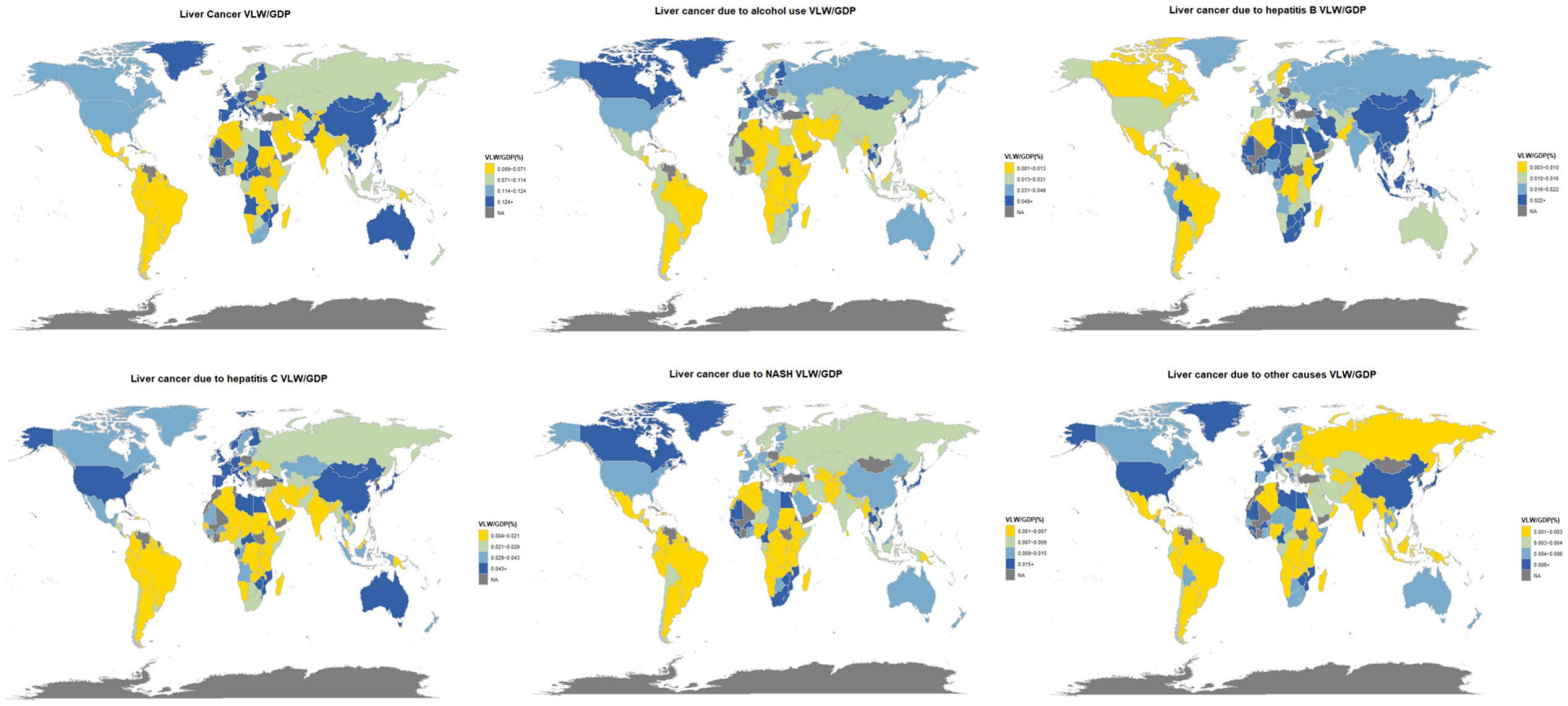
World heat maps of VLW/GDP in 2021 by country for liver cancer and its pathogenic factors. World map created using the ggmap package within RStudio statistical software (https://cran.r-project.org/web/packages/ggmap/index.html), licensed under GNU General Public License.

The nationwide distribution for liver cancer and its causative factors in 2021 are depicted in Figure 2, with the precise values detailed in Supplementary Table 1. To evaluate the robustness of our estimates to the income elasticity (IE) assumption, we recalculated VLW and VLW/GDP using the lower (IE = 0.55) and upper (IE = 1.5) bounds of the range reported in the literature (Supplementary Tables 2, 3). For example, in China the total VLW decreased from 80.8 billion USD (0.45% of GDP) under IE = 0.55 to 15.6 billion USD (0.09% of GDP) under IE = 1.5. Despite these differences in absolute amounts, the relative ranking of countries and regions remained largely stable across scenarios, indicating that our main conclusions about regional burden are robust to the choice of IE parameter.

## Discussion

The overall VLW attributed to liver cancer across 185 nations in 2021 is estimated at $141.95 B, representing 0.15% of the global GDP. Consistent with prior epidemiological studies, our findings underscore the substantial the worldwide impact of liver cancer, both in terms of health impact and economic welfare losses (31). The authors argue that these detailed worldwide, regional, and nation-specific projections provide essential evidence for enhancing location-specific distribution of resources, bolstering first-line prevention initiatives, and setting data-informed priorities for liver cancer management. Notable disparities exist in the economic burden across various super-regions. The proportion of VLW/GDP in the East Asia, Southeast Asia, and Oceania super-region is the highest (0.19%), followed closely by High-income super-region (0.16%), but the driving factors of the two are completely different.

The economic burden of liver cancer in the East Asia, Southeast Asia, and Oceania super-region mainly comes from HBV infection (VLW/GDP = 0.11%). The combined impact of these regions represents over 90% of global incidents of HBV-related liver cancer, with their contribution to etiology significantly surpassing that of other risk factors (32, 33). Furthermore, the early screening system for liver cancer is flawed, resulting in the majority of individuals receiving a diagnosis at a late stage (34, 35), further increasing the treatment cost and economic loss. The HBV vaccination rate is relatively high in high-income super-regions, and the economic burden of liver cancer mainly comes from HCV infection (VLW/GDP = 0.072%) and alcohol use (VLW/GDP = 0.042%). The primary route of transmission for HCV is through the use of injected drugs. The prevalence of HCV infections linked to injecting drug use has markedly risen in high-income nations (36). The sustained elevated levels of alcohol consumption in affluent regions, including Asia-Pacific and Europe, have resulted in a steady rise in deaths associated with alcohol-related liver cancer (8, 37). Furthermore, obesity affects many countries around the world and brings serious health problems to countless people (38). NASH shows an important increase globally, particularly in Australasia, Central Asia, and affluent North America, where DALYs rates have risen beyond threefold since 1990 (39). The cost of NASH-related liver cancer in affluent countries (VLW/GDP = 0.013%) is directly related to the increase in obesity rates (40, 41). In contrast, although the Sub-Saharan Africa super-region (VLW/GDP = 0.097%) and the South Asia super-region (VLW/GDP = 0.091%) bear a relatively high burden of liver cancer, their economic aggregates are relatively low, which leads to relatively small absolute VLW values (1.806 B and 3.626 B US dollars respectively). This “high relative burden-low absolute expenditure” model highlights the weak healthcare system in this region. For example, in Africa, only 18% of newborns received the first dose of the vaccine on time. This area represents 63% of the global incidence of new HBV infections (42). Besides, liver cancer screening services are also insufficient (43, 44). Moreover, it is noteworthy that Mongolia exhibits a VLW/GDP ratio of 1.028%—nearly three times higher than that of the next highest country, the Republic of Korea, which stands at 0.368%. This situation underscores underlying structural deficiencies in Mongolia, including institutional fragility and significant socio-economic externalities. To mitigate the elevated VLW/GDP ratio, Mongolia should pursue a strategic shift toward economic diversification, enhance public governance mechanisms, and allocate greater resources to evidence-based preventive medicine. Such integrated measures would contribute to a more efficient allocation of health resources, reduce the burden of disease, and ultimately promote sustainable welfare optimization.

The distribution of healthcare supplies, policy response skills, and the level of economic and social growth all exert influence on the economic burden of liver cancer, according to studies (45). In terms of health resource allocation, affluent nations have high-quality medical resources, such as easy access to antiviral medications and targeted therapy treatments for liver cancer, but medical treatment costs are expensive (46, 47). As a result, the VLW value remains high (the total VLW in affluent regions is 88 B US dollars), presenting a unique phenomenon of “high investment-high burden.” At the policy response level, South Korea has successfully increased the five-year success rate of hepatic carcinoma more than 40% by popularizing HBV screening and standardizing the ultrasound combined with AFP monitoring system (48), verifying the key role of the early screening policy in reducing the expense of advanced treatment. Regarding the advancement of societal and economic development, low-income nations face financial constraints, and there are major flaws in the control and prevention system of HBV (for example, only one-third of African countries provide free medical treatment services) (49), which makes it difficult for patients to receive standardized treatment and forms a vicious cycle of “low investment-high burden.”

The VLW/GDP indicator provides an innovative comprehensive measurement dimension for the assessment of the economic cost of illnesses is calculated by combining immediate healthcare costs and indirect productivity losses This indicator forms a complementary relationship with the traditional DALYs (Disability-Adjusted Life Years): DALYs mainly reflects the health loss caused by diseases, while VLW/GDP quantifies the impact of diseases on the national economy from an economic perspective (50). The DALYs of global liver cancer show a downward trend. This positive change may be attributed to the synergistic effect of multi-dimensional public health intervention measures, including comprehensive intervention measures such as optimized liver cancer screening, widespread vaccination, and improved accessibility of viral hepatitis treatment (51, 52). However, the economic burden revealed by the VLW/GDP indicator remains significant, highlights that the following key shortcomings in the current prevention and control system. First and foremost, just 45% of newborns globally receive the initial injection of HBV vaccine inside a day of delivery (42). It is far from achieving the elimination goal. Secondly, the coverage rate of antiviral treatment for hepatitis B patients is less than 20%, and the treatment gap is huge (49). Furthermore, key populations of HCV (such as injecting drug users) face systemic obstacles in treatment due to issues such as social discrimination, addiction challenges, and medical accessibility (53–56).

Based on the collaborative analysis of VLW/GDP and DALYs indicators, we propose suggestions for hierarchical prevention and control: in areas with a high prevalence of HBV, efforts should be focused on breaking through the dual bottlenecks of vaccination coverage and antiviral treatment coverage. In wealthy nations, it is critical to enhance the full-process management of HCV testing and management, particularly to address treatment barriers for marginalized populations. At the global level, it is necessary to jointly promote the control of alcohol consumption and the prevention and treatment of metabolic liver diseases. Meanwhile, drawing on the successful experience of South Korea in improving the survival rate of liver cancer through systematic early screening (ultrasound combined with AFP monitoring), a hierarchical medical treatment system is constructed. The successful model exemplified by South Korea demonstrates that strategic public health investment can serve as a powerful instrument for macroeconomic management. By proactively mitigating future welfare losses (value of lost welfare, VLW), such precision prevention strategies—grounded in targeted screening—enhance national economic resilience and improve overall population well-being. This outcome is directly reflected in the optimized VLW/GDP ratio, signifying a transformation of the healthcare system from a cost center into a strategic investment for national welfare and economic stability.

While super-regional analysis facilitates broad international comparisons, it may mask substantial within-region heterogeneity. For example, Mongolia exhibits a VLW/GDP ratio of 1.028%—nearly three times higher than the next highest country in the region (Republic of Korea 0.368%, Japan 0.352%). In Sub-Saharan Africa, the average VLW/GDP ratio is 0.097% but the absolute VLW is only 1.806 billion USD, reflecting weaker health systems and smaller economic aggregates. These country-level deviations highlight the importance of complementing super-regional estimates with national-level analyses when designing policy interventions. We also acknowledge that, although we used the most recent Global Burden of Disease data, DALY estimates for many low- and middle-income countries are modeled rather than based on direct surveillance, which may add further uncertainty to our regional estimates. Together, these factors suggest that our super-regional results should be interpreted as indicative rather than definitive and that future work using more granular, country-level data would be valuable.

The present investigation poses a number of constraints that should be weighed when analyzing its conclusions. To begin with, this study mainly estimates based on theoretical models. For example, the valuation of the VSL in various countries is modeled through empirical data from the United States and deduced by combining transformation methods. This method may be difficult to reflect regional data differences. Our base-case estimates assumed a unity income elasticity (IE = 1.0) for VSL transfers. Sensitivity analyses using the lower (IE = 0.55) and upper (IE = 1.5) bounds of the literature showed that while absolute VLW estimates varied markedly, for example, China’s total VLW decreased from 80.8 billion USD (0.45% of GDP) to 15.6 billion USD (0.09% of GDP), the relative ranking of regions and the identification of high-burden countries remained robust. This suggests that our main conclusions about the relative economic burden of liver cancer are not an artifact of a single parameter choice. To reduce the potential bias in WTP, while accounting for financial status and PPP to the greatest extent feasible, the study ultimately adopted 1.0 as the internal impact index. Second, the VSL age adjustment function *f*(*a*) is constructed based on the classical estimates of Chen et al. (15). These estimates may also fail to reflect regional differences, which to some extent limits the accuracy of this analysis. Finally, considering the scarcity of robust epidemiological study information on liver cancer in most countries, the DALY indicators in some countries in the GBD study inevitably contain a large proportion of simulation projections.

## Conclusion

This study provides one of the first global, super-regional, and country-level assessments of the macroeconomic impact of liver cancer using a welfare-based VLW framework. We find substantial welfare losses with marked cross-country heterogeneity, including extreme outliers such as Mongolia. Our results highlight both the heavy economic burden of liver cancer and the potential of cost-effective interventions to reduce it. Future work should refine these estimates with more granular data and evaluate the economic impact of specific preventive and control strategies to guide policy and investment.

## Data Availability

The original contributions presented in the study are included in the article/Supplementary material, further inquiries can be directed to the corresponding author.
