# Supplementary material for: The economic ramifications of liver cancer on a global, regional, and national scale
Source: Front Med (Lausanne). 2025 Nov 3;12:1694588. doi: 10.3389/fmed.2025.1694588 (PMC12622226; doi:10.3389/fmed.2025.1694588)
Supplement: Supplementary file 1 [file Data_Sheet_1.pdf]

Supplementary Table 1. VLW and VLW/GDP by nation in 2021 for liver cancer and its pathogenic factors, generated using IE of the VSL at 1.00. All $ values are in 2021 USD, PPP.

IE=1.0	Liver cancer overall		Liver cancer due to alcohol use		Liver cancer due to hepatitis B		Liver cancer due to hepatitis C		Liver cancer due to NASH		Liver cancer due to other causes	
Country	VLW ($billion)	VLW/GDP(%)	VLW ($billion)	VLW/GDP(%)	VLW ($billion)	VLW/GDP(%)	VLW ($billion)	VLW/GDP(%)	VLW ($billion)	VLW/GDP(%)	VLW($billion)	VLW/GDP(%)
China	34.437	0.193	3.874	0.022	21.444	0.120	7.700	0.043	2.255	0.013	1.728	0.010
Cambodia	0.047	0.127	0.009	0.024	0.013	0.035	0.010	0.027	0.003	0.009	0.001	0.003
Indonesia	1.347	0.114	0.159	0.013	0.261	0.022	0.368	0.031	0.104	0.009	0.030	0.003
Lao People's Democratic Republic	0.017	0.091	0.004	0.024	0.007	0.037	0.004	0.020	0.001	0.007	0.000	0.002
Malaysia	0.329	0.088	0.045	0.012	0.172	0.046	0.058	0.016	0.036	0.010	0.009	0.002
Maldives	0.003	0.053	0.001	0.011	0.001	0.022	0.001	0.013	0.000	0.005	0.000	0.001
Myanmar	0.051	0.077	0.007	0.011	0.017	0.025	0.015	0.023	0.004	0.006	0.001	0.002
Philippines	0.785	0.199	0.107	0.027	0.175	0.044	0.088	0.022	0.037	0.009	0.012	0.003
Sri Lanka	0.039	0.044	0.007	0.008	0.010	0.011	0.006	0.007	0.003	0.003	0.001	0.001
Thailand	1.208	0.239	0.382	0.075	0.476	0.094	0.198	0.039	0.131	0.026	0.027	0.005
Timor-Leste	0.002	0.057	0.000	0.008	0.001	0.016	0.000	0.011	0.000	0.004	0.000	0.001
Socialist Republic of Viet Nam	0.788	0.215	0.227	0.062	0.353	0.096	0.135	0.037	0.065	0.018	0.021	0.006
Fiji	0.005	0.111	0.001	0.012	0.002	0.048	0.001	0.021	0.000	0.011	0.000	0.004
Kiribati	0.000	0.093	0.000	0.007	0.000	0.040	0.000	0.019	0.000	0.009	0.000	0.004
Marshall Islands	0.000	0.116	0.000	0.009	0.000	0.040	0.000	0.015	0.000	0.007	0.000	0.004
Federated States of Micronesia	0.000	0.070	0.000	0.008	0.000	0.035	0.000	0.012	0.000	0.007	0.000	0.003

Papua New Guinea	0.008	0.029	0.001	0.003	0.004	0.017	0.002	0.006	0.001	0.002	0.000	0.001
Samoa	0.001	0.166	0.000	0.008	0.000	0.033	0.000	0.013	0.000	0.007	0.000	0.002
Solomon Islands	0.001	0.054	0.000	0.006	0.000	0.029	0.000	0.008	0.000	0.004	0.000	0.002
Tonga	0.001	0.281	0.000	0.036	0.001	0.159	0.000	0.063	0.000	0.035	0.000	0.011
Vanuatu	0.001	0.057	0.000	0.006	0.000	0.028	0.000	0.010	0.000	0.005	0.000	0.002
Armenia	0.030	0.218	0.007	0.051	0.006	0.040	0.013	0.094	0.003	0.022	0.001	0.006
Azerbaijan	0.099	0.180	0.028	0.050	0.022	0.040	0.036	0.066	0.009	0.016	0.003	0.005
Georgia	0.029	0.151	0.007	0.038	0.005	0.028	0.009	0.050	0.002	0.012	0.001	0.003
Kazakhstan	0.187	0.095	0.051	0.026	0.040	0.020	0.069	0.035	0.018	0.009	0.006	0.003
Kyrgyzstan	0.005	0.053	0.001	0.013	0.001	0.013	0.002	0.022	0.000	0.004	0.000	0.002
Mongolia	0.157	1.028	0.038	0.248	0.041	0.270	0.070	0.461	0.011	0.073	0.005	0.030
Tajikistan	0.005	0.057	0.001	0.011	0.001	0.010	0.002	0.026	0.000	0.004	0.000	0.002
Turkmenistan	0.031	0.062	0.008	0.016	0.008	0.016	0.012	0.024	0.003	0.006	0.001	0.002
Uzbekistan	0.102	0.133	0.012	0.016	0.013	0.017	0.020	0.026	0.004	0.006	0.002	0.002
Albania	0.040	0.221	0.014	0.079	0.010	0.053	0.011	0.060	0.005	0.025	0.001	0.007
Bosnia and Herzegovina	0.054	0.229	0.021	0.088	0.011	0.049	0.014	0.061	0.006	0.027	0.002	0.007
Bulgaria	0.125	0.148	0.057	0.068	0.024	0.028	0.028	0.033	0.012	0.014	0.003	0.004
Croatia	0.080	0.116	0.039	0.056	0.019	0.028	0.010	0.015	0.010	0.015	0.003	0.004
Czech Republic	0.273	0.094	0.139	0.048	0.047	0.016	0.058	0.020	0.026	0.009	0.007	0.002
Hungary	0.121	0.066	0.051	0.028	0.026	0.014	0.027	0.015	0.013	0.007	0.003	0.002
The former Yugoslav Republic of Macedonia	0.040	0.286	0.016	0.117	0.009	0.064	0.010	0.071	0.004	0.031	0.001	0.009

Montenegro	0.012	0.199	0.005	0.079	0.003	0.043	0.003	0.050	0.001	0.024	0.000	0.006
Romania	0.323	0.113	0.143	0.050	0.064	0.022	0.076	0.027	0.033	0.011	0.009	0.003
Serbia	0.174	0.263	0.055	0.083	0.027	0.041	0.072	0.108	0.014	0.022	0.004	0.006
Slovakia	0.151	0.125	0.070	0.058	0.030	0.025	0.036	0.029	0.015	0.012	0.004	0.004
Slovenia	0.096	0.157	0.038	0.062	0.020	0.032	0.025	0.040	0.011	0.018	0.003	0.004
Belarus	0.070	0.101	0.025	0.035	0.013	0.019	0.019	0.027	0.006	0.008	0.002	0.003
Estonia	0.045	0.122	0.018	0.050	0.008	0.020	0.014	0.038	0.005	0.013	0.001	0.003
Latvia	0.046	0.120	0.020	0.052	0.007	0.019	0.014	0.036	0.005	0.012	0.001	0.003
Lithuania	0.085	0.126	0.032	0.047	0.015	0.022	0.025	0.037	0.008	0.011	0.002	0.003
Republic of Moldova	0.019	0.141	0.008	0.056	0.004	0.028	0.006	0.042	0.002	0.012	0.001	0.004
Russian Federation	1.539	0.083	0.577	0.031	0.300	0.016	0.475	0.026	0.153	0.008	0.047	0.003
Ukraine	0.096	0.048	0.032	0.016	0.022	0.011	0.028	0.014	0.008	0.004	0.003	0.001
Brunei Darussalam	0.018	0.126	0.002	0.011	0.009	0.064	0.006	0.044	0.001	0.009	0.001	0.005
Japan	17.725	0.352	1.734	0.034	1.715	0.034	13.116	0.261	0.852	0.017	0.420	0.008
Republic of Korea	6.695	0.368	1.165	0.064	3.759	0.207	1.312	0.072	0.503	0.028	0.204	0.011
Singapore	0.543	0.125	0.039	0.009	0.318	0.073	0.138	0.032	0.030	0.007	0.013	0.003
Australia	1.934	0.124	0.761	0.049	0.233	0.015	0.678	0.044	0.229	0.015	0.083	0.005
New Zealand	0.232	0.091	0.088	0.035	0.031	0.012	0.077	0.030	0.028	0.011	0.011	0.004
Andorra	0.010	0.305	0.003	0.096	0.001	0.035	0.005	0.140	0.001	0.028	0.000	0.015
Austria	0.702	0.146	0.385	0.080	0.040	0.008	0.220	0.046	0.046	0.010	0.020	0.004
Belgium	0.736	0.123	0.258	0.043	0.068	0.011	0.340	0.057	0.052	0.009	0.027	0.004
Cyprus	0.034	0.114	0.012	0.040	0.004	0.014	0.014	0.046	0.002	0.008	0.001	0.005

Denmark	0.375	0.092	0.144	0.035	0.047	0.012	0.144	0.035	0.027	0.007	0.014	0.003
Finland	0.457	0.155	0.155	0.053	0.055	0.019	0.196	0.067	0.036	0.012	0.018	0.006
France	5.394	0.182	1.591	0.054	0.597	0.020	2.638	0.089	0.425	0.014	0.209	0.007
Germany	6.480	0.149	2.782	0.064	0.582	0.013	2.272	0.052	0.531	0.012	0.308	0.007
Greece	0.316	0.145	0.091	0.042	0.110	0.050	0.081	0.037	0.027	0.012	0.013	0.006
Iceland	0.025	0.097	0.009	0.034	0.003	0.013	0.010	0.038	0.002	0.008	0.001	0.004
Ireland	0.435	0.082	0.156	0.029	0.049	0.009	0.178	0.033	0.037	0.007	0.018	0.003
Israel	0.227	0.046	0.059	0.012	0.032	0.006	0.107	0.022	0.021	0.004	0.011	0.002
Italy	3.553	0.163	0.840	0.039	0.321	0.015	2.132	0.098	0.175	0.008	0.077	0.004
Luxembourg	0.089	0.104	0.033	0.039	0.010	0.012	0.037	0.043	0.007	0.008	0.004	0.004
Malta	0.015	0.077	0.006	0.028	0.002	0.010	0.006	0.031	0.001	0.006	0.001	0.003
Netherlands	0.860	0.082	0.359	0.034	0.114	0.011	0.275	0.026	0.084	0.008	0.041	0.004
Norway	0.481	0.096	0.153	0.030	0.058	0.012	0.218	0.043	0.037	0.007	0.022	0.004
Portugal	0.428	0.167	0.174	0.068	0.051	0.020	0.165	0.064	0.031	0.012	0.016	0.006
Spain	2.360	0.162	0.679	0.046	0.193	0.013	1.300	0.089	0.147	0.010	0.082	0.006
Sweden	0.562	0.088	0.233	0.037	0.038	0.006	0.218	0.034	0.050	0.008	0.028	0.004
Switzerland	0.979	0.120	0.363	0.045	0.124	0.015	0.389	0.048	0.069	0.008	0.036	0.004
United Kingdom of Great Britain and Northern Ireland	5.254	0.167	1.839	0.059	0.638	0.020	2.203	0.070	0.441	0.014	0.215	0.007
Argentina	0.118	0.024	0.034	0.007	0.023	0.005	0.045	0.009	0.012	0.002	0.007	0.001
Chile	0.205	0.065	0.061	0.019	0.033	0.010	0.079	0.025	0.024	0.008	0.010	0.003
Uruguay	0.042	0.068	0.011	0.019	0.007	0.011	0.014	0.022	0.004	0.006	0.002	0.003
Canada	2.445	0.122	1.228	0.061	0.152	0.008	0.587	0.029	0.393	0.020	0.115	0.006

United States of America	28.243	0.119	8.371	0.035	3.051	0.013	11.331	0.048	3.220	0.014	2.355	0.010
Antigua and Barbuda	0.001	0.052	0.000	0.018	0.000	0.012	0.000	0.013	0.000	0.005	0.000	0.002
Commonwealth of the Bahamas	0.006	0.053	0.002	0.018	0.002	0.015	0.002	0.013	0.001	0.006	0.000	0.003
Barbados	0.005	0.086	0.002	0.030	0.001	0.015	0.001	0.024	0.001	0.011	0.000	0.004
Belize	0.001	0.035	0.000	0.010	0.000	0.009	0.000	0.010	0.000	0.004	0.000	0.002
Dominica	0.001	0.095	0.000	0.033	0.000	0.020	0.000	0.027	0.000	0.012	0.000	0.004
Dominican Republic	0.029	0.030	0.009	0.010	0.008	0.008	0.008	0.008	0.003	0.003	0.002	0.002
Grenada	0.001	0.061	0.000	0.024	0.000	0.014	0.000	0.016	0.000	0.006	0.000	0.003
Guyana	0.004	0.044	0.001	0.013	0.001	0.010	0.001	0.011	0.000	0.004	0.000	0.002
Haiti	0.004	0.021	0.001	0.005	0.001	0.006	0.001	0.006	0.000	0.002	0.000	0.001
Jamaica	0.006	0.039	0.002	0.011	0.001	0.009	0.002	0.012	0.001	0.005	0.000	0.002
Saint Lucia	0.001	0.049	0.000	0.018	0.000	0.010	0.000	0.012	0.000	0.005	0.000	0.002
Saint Vincent and the Grenadines	0.001	0.074	0.000	0.029	0.000	0.018	0.000	0.018	0.000	0.007	0.000	0.003
Suriname	0.002	0.051	0.001	0.017	0.000	0.014	0.000	0.013	0.000	0.005	0.000	0.003
Trinidad and Tobago	0.014	0.058	0.005	0.019	0.003	0.013	0.004	0.017	0.002	0.008	0.001	0.003
Plurinational State of Bolivia	0.026	0.064	0.007	0.018	0.009	0.023	0.004	0.009	0.003	0.008	0.002	0.004
Ecuador	0.056	0.052	0.013	0.012	0.019	0.018	0.008	0.007	0.008	0.007	0.003	0.003
Peru	0.161	0.071	0.032	0.014	0.039	0.017	0.015	0.007	0.014	0.006	0.006	0.003

Colombia	0.143	0.045	0.042	0.013	0.027	0.008	0.048	0.015	0.020	0.006	0.008	0.002
Costa Rica	0.064	0.099	0.019	0.029	0.009	0.013	0.028	0.042	0.008	0.012	0.003	0.005
El Salvador	0.010	0.036	0.002	0.008	0.001	0.005	0.005	0.016	0.001	0.004	0.001	0.002
Guatemala	0.067	0.078	0.015	0.017	0.009	0.011	0.031	0.035	0.007	0.008	0.004	0.004
Honduras	0.014	0.050	0.003	0.011	0.002	0.007	0.007	0.025	0.002	0.005	0.001	0.003
Mexico	0.875	0.066	0.200	0.015	0.065	0.005	0.434	0.033	0.080	0.006	0.034	0.003
Nicaragua	0.007	0.053	0.002	0.012	0.001	0.007	0.003	0.022	0.001	0.005	0.000	0.003
Panama	0.034	0.050	0.010	0.015	0.005	0.007	0.014	0.021	0.004	0.006	0.002	0.003
Brazil	0.672	0.040	0.201	0.012	0.126	0.008	0.243	0.015	0.058	0.003	0.030	0.002
Paraguay	0.024	0.060	0.008	0.021	0.004	0.011	0.008	0.021	0.002	0.005	0.001	0.003
Algeria	0.054	0.029	0.005	0.002	0.016	0.009	0.019	0.010	0.006	0.003	0.003	0.002
Bahrain	0.017	0.040	0.002	0.004	0.006	0.015	0.006	0.015	0.002	0.006	0.001	0.002
Egypt	1.066	0.251	0.099	0.023	0.135	0.032	0.661	0.156	0.133	0.031	0.061	0.014
Islamic Republic of Iran	0.186	0.049	0.015	0.004	0.085	0.022	0.044	0.012	0.032	0.008	0.013	0.003
Iraq	0.112	0.054	0.011	0.005	0.039	0.019	0.040	0.019	0.014	0.007	0.007	0.003
Jordan	0.009	0.018	0.001	0.002	0.003	0.007	0.003	0.007	0.001	0.003	0.001	0.001
Kuwait	0.020	0.014	0.002	0.001	0.006	0.004	0.007	0.005	0.003	0.002	0.001	0.001
Lebanon	0.014	0.062	0.001	0.004	0.005	0.020	0.003	0.014	0.001	0.005	0.000	0.002
Libya	0.037	0.106	0.003	0.008	0.013	0.037	0.015	0.044	0.005	0.014	0.002	0.006
Morocco	0.013	0.009	0.001	0.001	0.005	0.003	0.005	0.004	0.002	0.001	0.001	0.001
Oman	0.026	0.030	0.002	0.003	0.011	0.012	0.008	0.009	0.003	0.004	0.001	0.002
Qatar	0.127	0.071	0.012	0.007	0.050	0.028	0.036	0.020	0.019	0.011	0.006	0.004
Saudi Arabia	0.505	0.058	0.026	0.003	0.204	0.023	0.172	0.020	0.085	0.010	0.027	0.003
Syrian Arab Republic	0.009	0.064	0.001	0.005	0.003	0.020	0.004	0.025	0.001	0.007	0.000	0.003

Turkey	0.511	0.062	0.076	0.009	0.208	0.025	0.129	0.016	0.063	0.008	0.026	0.003
United Arab Emirates	0.287	0.069	0.034	0.008	0.138	0.033	0.086	0.021	0.044	0.011	0.017	0.004
Afghanistan	0.015	0.107	0.000	0.002	0.002	0.014	0.002	0.014	0.001	0.004	0.000	0.003
Bangladesh	0.148	0.036	0.027	0.007	0.043	0.010	0.055	0.013	0.013	0.003	0.004	0.001
Bhutan	0.002	0.063	0.000	0.014	0.000	0.017	0.001	0.021	0.000	0.007	0.000	0.002
India	2.187	0.069	0.443	0.014	0.580	0.018	0.333	0.010	0.227	0.007	0.050	0.002
Nepal	0.034	0.092	0.005	0.014	0.005	0.015	0.006	0.017	0.002	0.006	0.001	0.002
Pakistan	1.255	0.360	0.022	0.006	0.023	0.007	0.086	0.025	0.012	0.003	0.005	0.002
Angola	0.085	0.127	0.007	0.011	0.011	0.017	0.020	0.029	0.003	0.005	0.002	0.002
Central African Republic	0.022	0.867	0.000	0.013	0.001	0.026	0.001	0.044	0.000	0.006	0.000	0.004
Congo	0.012	0.080	0.002	0.012	0.003	0.018	0.005	0.037	0.001	0.007	0.000	0.003
Democratic Republic of the Congo	0.020	0.037	0.002	0.004	0.004	0.007	0.009	0.016	0.001	0.002	0.001	0.001
Equatorial Guinea	0.006	0.046	0.001	0.006	0.001	0.009	0.002	0.016	0.000	0.004	0.000	0.001
Gabon	0.018	0.091	0.003	0.017	0.004	0.020	0.008	0.043	0.002	0.009	0.001	0.003
Burundi	0.004	0.131	0.000	0.004	0.000	0.007	0.000	0.008	0.000	0.003	0.000	0.002
Djibouti	0.002	0.071	0.000	0.009	0.001	0.018	0.001	0.016	0.000	0.007	0.000	0.003
Ethiopia	0.023	0.021	0.003	0.003	0.006	0.005	0.008	0.007	0.003	0.002	0.001	0.001
Kenya	0.063	0.058	0.010	0.009	0.010	0.009	0.014	0.013	0.008	0.007	0.003	0.003
Madagascar	0.010	0.066	0.001	0.004	0.001	0.007	0.001	0.007	0.000	0.003	0.000	0.002
Malawi	0.006	0.045	0.001	0.008	0.002	0.014	0.001	0.011	0.001	0.005	0.000	0.003
Mauritius	0.002	0.019	0.000	0.004	0.001	0.007	0.001	0.005	0.000	0.002	0.000	0.001

Mozambique	0.065	0.401	0.006	0.037	0.012	0.074	0.007	0.045	0.004	0.024	0.002	0.010
Rwanda	0.007	0.066	0.001	0.010	0.001	0.011	0.002	0.014	0.001	0.006	0.000	0.003
Seychelles	0.001	0.094	0.000	0.024	0.001	0.039	0.000	0.022	0.000	0.011	0.000	0.002
Somalia	0.009	0.097	0.001	0.010	0.003	0.027	0.002	0.021	0.001	0.009	0.000	0.005
United Republic of Tanzania	0.071	0.100	0.006	0.008	0.008	0.012	0.008	0.012	0.004	0.006	0.002	0.003
Uganda	0.022	0.054	0.005	0.012	0.006	0.014	0.005	0.012	0.002	0.006	0.001	0.003
Zambia	0.014	0.063	0.002	0.008	0.003	0.012	0.003	0.012	0.001	0.006	0.001	0.003
Botswana	0.017	0.091	0.003	0.017	0.006	0.033	0.004	0.021	0.002	0.011	0.001	0.004
Lesotho	0.005	0.187	0.001	0.036	0.002	0.081	0.001	0.043	0.001	0.022	0.000	0.009
Namibia	0.009	0.069	0.001	0.008	0.002	0.013	0.001	0.009	0.001	0.004	0.000	0.002
South Africa	0.505	0.120	0.102	0.024	0.160	0.038	0.107	0.025	0.064	0.015	0.019	0.005
Kingdom of Eswatini	0.016	0.336	0.002	0.047	0.005	0.105	0.002	0.043	0.001	0.028	0.000	0.010
Zimbabwe	0.043	0.158	0.006	0.021	0.014	0.052	0.013	0.048	0.005	0.018	0.002	0.009
Benin	0.021	0.121	0.003	0.018	0.010	0.055	0.004	0.020	0.002	0.014	0.001	0.006
Burkina Faso	0.036	0.181	0.008	0.040	0.017	0.087	0.006	0.031	0.004	0.019	0.002	0.008
Cameroon	0.079	0.175	0.012	0.026	0.029	0.065	0.010	0.021	0.008	0.018	0.003	0.007
Republic of Cabo Verde	0.004	0.211	0.001	0.040	0.002	0.092	0.001	0.047	0.001	0.034	0.000	0.010
Chad	0.045	0.380	0.002	0.018	0.007	0.056	0.002	0.018	0.001	0.010	0.001	0.005
Cote d'Ivoire	0.027	0.038	0.005	0.007	0.012	0.017	0.004	0.005	0.003	0.004	0.001	0.002
Republic of the Gambia	0.006	0.293	0.001	0.050	0.003	0.154	0.001	0.040	0.001	0.032	0.000	0.014
Ghana	0.078	0.098	0.013	0.016	0.036	0.045	0.012	0.015	0.009	0.011	0.003	0.004
Guinea	0.120	0.703	0.004	0.026	0.016	0.093	0.006	0.033	0.004	0.021	0.002	0.010

Guinea-Bissau	0.025	1.340	0.000	0.027	0.002	0.089	0.000	0.022	0.000	0.016	0.000	0.008
Liberia	0.006	0.175	0.001	0.031	0.003	0.090	0.001	0.025	0.001	0.020	0.000	0.008
Mauritania	0.019	0.201	0.003	0.028	0.009	0.102	0.003	0.035	0.003	0.030	0.001	0.009
Niger	0.016	0.110	0.002	0.011	0.005	0.033	0.002	0.016	0.001	0.009	0.001	0.004
Nigeria	0.236	0.054	0.036	0.008	0.078	0.018	0.047	0.011	0.029	0.006	0.011	0.002
Sao Tome and Principe	0.000	0.026	0.000	0.005	0.000	0.013	0.000	0.004	0.000	0.003	0.000	0.001
Senegal	0.027	0.098	0.004	0.014	0.014	0.050	0.004	0.013	0.004	0.016	0.002	0.006
Togo	0.009	0.107	0.001	0.013	0.003	0.038	0.001	0.017	0.001	0.011	0.000	0.005
American Samoa	0.001	0.119	0.000	0.012	0.000	0.060	0.000	0.024	0.000	0.017	0.000	0.005
Bermuda	0.003	0.047	0.001	0.019	0.001	0.009	0.001	0.013	0.000	0.006	0.000	0.002
Greenland	0.005	0.140	0.002	0.058	0.001	0.016	0.001	0.041	0.001	0.017	0.000	0.010
Guam	0.006	0.103	0.001	0.015	0.004	0.060	0.001	0.019	0.001	0.012	0.000	0.003
Principality of Monaco	0.023	0.270	0.008	0.090	0.003	0.032	0.010	0.115	0.002	0.026	0.001	0.011
Republic of Nauru	0.000	0.064	0.000	0.009	0.000	0.041	0.000	0.011	0.000	0.006	0.000	0.003
Northern Mariana Islands	0.001	0.136	0.000	0.016	0.001	0.065	0.000	0.023	0.000	0.016	0.000	0.004
Republic of Palau	0.000	0.181	0.000	0.031	0.000	0.120	0.000	0.027	0.000	0.019	0.000	0.005
Puerto Rico	0.095	0.089	0.030	0.028	0.020	0.019	0.025	0.023	0.013	0.012	0.004	0.004
Saint Kitts and Nevis	0.001	0.108	0.000	0.035	0.000	0.027	0.000	0.027	0.000	0.011	0.000	0.005

Republic of San Marino	0.001	0.079	0.001	0.030	0.000	0.009	0.001	0.032	0.000	0.006	0.000	0.003
Tuvalu	0.000	0.097	0.000	0.012	0.000	0.052	0.000	0.022	0.000	0.011	0.000	0.004
United States Virgin Islands	0.004	0.080	0.001	0.031	0.001	0.018	0.001	0.020	0.000	0.011	0.000	0.003
Sudan	0.012	0.035	0.001	0.004	0.004	0.010	0.003	0.010	0.001	0.004	0.001	0.003

Supplementary Table 2. VLW and VLW/GDP by nation in 2021 for liver cancer and its pathogenic factors, generated using IE of the VSL at 0.55. All $ values are in 2021 USD, PPP.

IE=0.55	Liver cancer overall		Liver cancer due to alcohol use		Liver cancer due to hepatitis B		Liver cancer due to hepatitis C		Liver cancer due to NASH		Liver cancer due to other causes	
Country	VLW($billion)	VLW/GDP(%)	VLW($billion)	VLW/GDP(%)	VLW($billion)	VLW/GDP(%)	VLW($billion)	VLW/GDP(%)	VLW($billion)	VLW/GDP(%)	VLW($billion)	VLW/GDP(%)
China	80.815	0.453	8.446	0.047	46.752	0.262	16.787	0.094	4.915	0.028	3.768	0.021
Cambodia	0.172	0.469	0.042	0.113	0.062	0.168	0.047	0.128	0.016	0.042	0.005	0.014
Indonesia	3.290	0.277	0.565	0.048	0.926	0.078	1.305	0.110	0.369	0.031	0.106	0.009
Lao People's Democratic Republic	0.078	0.414	0.020	0.107	0.032	0.169	0.017	0.089	0.006	0.033	0.002	0.011
Malaysia	0.746	0.200	0.105	0.028	0.400	0.107	0.135	0.036	0.084	0.022	0.020	0.005
Maldives	0.007	0.126	0.001	0.026	0.003	0.053	0.002	0.031	0.001	0.013	0.000	0.003
Myanmar	0.278	0.419	0.045	0.067	0.105	0.158	0.095	0.144	0.024	0.036	0.007	0.011
Philippines	1.643	0.417	0.415	0.105	0.681	0.173	0.344	0.087	0.145	0.037	0.046	0.012
Sri Lanka	0.098	0.111	0.025	0.028	0.037	0.042	0.024	0.027	0.010	0.012	0.003	0.003
Thailand	3.445	0.680	1.082	0.214	1.349	0.266	0.560	0.111	0.372	0.073	0.078	0.015
Timor-Leste	0.006	0.177	0.001	0.033	0.002	0.069	0.002	0.048	0.001	0.018	0.000	0.006
Socialist Republic of Viet Nam	3.043	0.830	0.859	0.234	1.334	0.364	0.511	0.139	0.245	0.067	0.078	0.021
Fiji	0.014	0.330	0.002	0.041	0.007	0.164	0.003	0.073	0.002	0.039	0.001	0.013
Kiribati	0.001	0.378	0.000	0.033	0.001	0.190	0.000	0.091	0.000	0.042	0.000	0.020

Marshall Islands	0.001	0.223	0.000	0.026	0.000	0.120	0.000	0.044	0.000	0.021	0.000	0.010
Federated States of Micronesia	0.001	0.250	0.000	0.029	0.001	0.135	0.000	0.048	0.000	0.027	0.000	0.011
Papua New Guinea	0.035	0.135	0.004	0.015	0.019	0.074	0.007	0.026	0.003	0.011	0.002	0.006
Samoa	0.002	0.234	0.000	0.030	0.001	0.120	0.000	0.048	0.000	0.025	0.000	0.009
Solomon Islands	0.004	0.246	0.000	0.028	0.002	0.145	0.001	0.041	0.000	0.020	0.000	0.011
Tonga	0.005	1.019	0.001	0.121	0.003	0.531	0.001	0.209	0.001	0.117	0.000	0.036
Vanuatu	0.002	0.211	0.000	0.024	0.001	0.115	0.000	0.043	0.000	0.020	0.000	0.009
Armenia	0.101	0.727	0.024	0.173	0.019	0.137	0.045	0.321	0.010	0.075	0.003	0.021
Azerbaijan	0.311	0.567	0.088	0.161	0.069	0.127	0.116	0.211	0.027	0.050	0.009	0.017
Georgia	0.081	0.429	0.024	0.126	0.017	0.090	0.031	0.164	0.007	0.038	0.002	0.011
Kazakhstan	0.450	0.228	0.125	0.063	0.098	0.050	0.168	0.085	0.043	0.022	0.014	0.007
Kyrgyzstan	0.029	0.318	0.007	0.078	0.007	0.075	0.012	0.129	0.002	0.025	0.001	0.010
Mongolia	0.575	3.762	0.131	0.857	0.143	0.934	0.244	1.594	0.039	0.253	0.016	0.105
Tajikistan	0.035	0.388	0.007	0.079	0.007	0.073	0.017	0.187	0.002	0.026	0.001	0.015
Turkmenistan	0.091	0.182	0.023	0.045	0.022	0.044	0.034	0.069	0.008	0.017	0.003	0.006
Uzbekistan	0.249	0.321	0.058	0.075	0.063	0.081	0.096	0.124	0.021	0.027	0.009	0.011
Albania	0.120	0.665	0.042	0.235	0.028	0.156	0.032	0.176	0.013	0.075	0.004	0.021
Bosnia and Herzegovina	0.153	0.647	0.058	0.245	0.032	0.135	0.040	0.170	0.018	0.076	0.005	0.020
Bulgaria	0.274	0.325	0.126	0.150	0.052	0.062	0.062	0.073	0.027	0.031	0.007	0.009
Croatia	0.151	0.218	0.072	0.104	0.036	0.052	0.019	0.028	0.019	0.027	0.005	0.007

Czech Republic	0.424	0.146	0.213	0.073	0.072	0.025	0.089	0.031	0.040	0.014	0.010	0.003
Hungary	0.218	0.120	0.093	0.051	0.048	0.026	0.049	0.027	0.023	0.013	0.006	0.003
The former Yugoslav Republic of Macedonia	0.112	0.797	0.045	0.319	0.025	0.176	0.027	0.193	0.012	0.085	0.003	0.024
Montenegro	0.029	0.503	0.011	0.195	0.006	0.107	0.007	0.125	0.003	0.060	0.001	0.016
Romania	0.656	0.229	0.288	0.101	0.130	0.045	0.153	0.053	0.066	0.023	0.018	0.006
Serbia	0.422	0.638	0.134	0.203	0.067	0.101	0.176	0.266	0.035	0.053	0.010	0.015
Slovakia	0.262	0.218	0.119	0.098	0.051	0.042	0.060	0.050	0.025	0.021	0.007	0.006
Slovenia	0.144	0.234	0.057	0.093	0.029	0.048	0.037	0.060	0.016	0.027	0.004	0.006
Belarus	0.179	0.257	0.068	0.098	0.037	0.053	0.053	0.075	0.016	0.023	0.005	0.007
Estonia	0.071	0.190	0.028	0.076	0.012	0.031	0.022	0.058	0.007	0.020	0.002	0.005
Latvia	0.082	0.216	0.035	0.092	0.013	0.034	0.024	0.063	0.008	0.021	0.002	0.006
Lithuania	0.133	0.198	0.052	0.078	0.024	0.035	0.041	0.061	0.012	0.019	0.004	0.005
Republic of Moldova	0.063	0.462	0.025	0.181	0.012	0.089	0.019	0.136	0.006	0.040	0.002	0.013
Russian Federation	3.407	0.185	1.261	0.068	0.657	0.036	1.038	0.056	0.334	0.018	0.102	0.006
Ukraine	0.315	0.158	0.107	0.053	0.075	0.038	0.095	0.047	0.028	0.014	0.010	0.005
Brunei Darussalam	0.027	0.194	0.002	0.016	0.013	0.093	0.009	0.065	0.002	0.012	0.001	0.007
Japan	23.131	0.459	2.248	0.045	2.223	0.044	17.004	0.338	1.105	0.022	0.544	0.011
Republic of Korea	9.552	0.525	1.603	0.088	5.169	0.284	1.805	0.099	0.692	0.038	0.281	0.015
Singapore	0.528	0.122	0.037	0.009	0.303	0.070	0.132	0.030	0.029	0.007	0.013	0.003

Australia	2.139	0.137	0.819	0.053	0.250	0.016	0.730	0.047	0.247	0.016	0.089	0.006
New Zealand	0.277	0.109	0.104	0.041	0.037	0.015	0.091	0.036	0.032	0.013	0.013	0.005
Andorra	0.013	0.396	0.004	0.122	0.001	0.045	0.006	0.176	0.001	0.035	0.001	0.018
Austria	0.809	0.168	0.438	0.091	0.045	0.009	0.250	0.052	0.052	0.011	0.023	0.005
Belgium	0.864	0.144	0.299	0.050	0.079	0.013	0.394	0.066	0.060	0.010	0.031	0.005
Cyprus	0.048	0.158	0.017	0.057	0.006	0.019	0.019	0.064	0.003	0.011	0.002	0.006
Denmark	0.380	0.093	0.145	0.036	0.047	0.012	0.146	0.036	0.027	0.007	0.014	0.003
Finland	0.525	0.179	0.178	0.060	0.063	0.021	0.224	0.076	0.042	0.014	0.020	0.007
France	6.813	0.230	1.982	0.067	0.744	0.025	3.288	0.111	0.530	0.018	0.261	0.009
Germany	7.453	0.171	3.200	0.074	0.670	0.015	2.613	0.060	0.611	0.014	0.354	0.008
Greece	0.563	0.258	0.159	0.073	0.193	0.088	0.142	0.065	0.047	0.021	0.022	0.010
Iceland	0.026	0.099	0.009	0.035	0.003	0.014	0.010	0.038	0.002	0.008	0.001	0.004
Ireland	0.367	0.069	0.131	0.025	0.041	0.008	0.149	0.028	0.031	0.006	0.015	0.003
Israel	0.266	0.054	0.067	0.014	0.036	0.007	0.124	0.025	0.025	0.005	0.013	0.003
Italy	4.774	0.219	1.131	0.052	0.432	0.020	2.870	0.132	0.235	0.011	0.104	0.005
Luxembourg	0.068	0.080	0.025	0.029	0.007	0.009	0.028	0.032	0.005	0.006	0.003	0.003
Malta	0.020	0.103	0.007	0.038	0.003	0.013	0.008	0.041	0.002	0.008	0.001	0.004
Netherlands	0.943	0.089	0.387	0.037	0.123	0.012	0.297	0.028	0.091	0.009	0.044	0.004
Norway	0.435	0.086	0.135	0.027	0.052	0.010	0.194	0.038	0.033	0.007	0.020	0.004
Portugal	0.705	0.275	0.281	0.110	0.083	0.032	0.266	0.104	0.049	0.019	0.026	0.010
Spain	3.506	0.240	0.991	0.068	0.282	0.019	1.896	0.130	0.215	0.015	0.119	0.008
Sweden	0.608	0.095	0.249	0.039	0.041	0.006	0.234	0.037	0.054	0.008	0.030	0.005
Switzerland	0.869	0.107	0.322	0.040	0.110	0.014	0.345	0.042	0.061	0.007	0.032	0.004
United Kingdom of Great Britain	6.452	0.205	2.221	0.071	0.771	0.025	2.660	0.085	0.533	0.017	0.259	0.008

and Northern Ireland												
Argentina	0.283	0.058	0.080	0.017	0.053	0.011	0.106	0.022	0.028	0.006	0.015	0.003
Chile	0.405	0.128	0.120	0.038	0.064	0.020	0.154	0.049	0.046	0.015	0.020	0.006
Uruguay	0.070	0.115	0.021	0.035	0.013	0.021	0.025	0.042	0.007	0.011	0.003	0.006
Canada	2.846	0.142	1.410	0.070	0.174	0.009	0.674	0.034	0.451	0.022	0.132	0.007
United States of America	28.402	0.120	8.371	0.035	3.051	0.013	11.331	0.048	3.220	0.014	2.355	0.010
Antigua and Barbuda	0.002	0.094	0.001	0.034	0.000	0.022	0.000	0.024	0.000	0.010	0.000	0.004
Commonwealth of the Bahamas	0.010	0.084	0.003	0.028	0.003	0.023	0.002	0.020	0.001	0.009	0.000	0.004
Barbados	0.008	0.154	0.003	0.055	0.001	0.028	0.002	0.044	0.001	0.020	0.000	0.007
Belize	0.003	0.109	0.001	0.031	0.001	0.028	0.001	0.029	0.000	0.012	0.000	0.007
Dominica	0.001	0.254	0.000	0.087	0.000	0.054	0.000	0.070	0.000	0.031	0.000	0.011
Dominican Republic	0.076	0.081	0.024	0.025	0.020	0.021	0.020	0.021	0.008	0.008	0.004	0.004
Grenada	0.002	0.156	0.001	0.060	0.000	0.034	0.000	0.039	0.000	0.016	0.000	0.007
Guyana	0.008	0.099	0.002	0.031	0.002	0.025	0.002	0.026	0.001	0.010	0.000	0.006
Haiti	0.023	0.111	0.006	0.028	0.006	0.029	0.007	0.033	0.002	0.009	0.002	0.008
Jamaica	0.019	0.129	0.005	0.037	0.004	0.029	0.006	0.040	0.002	0.017	0.001	0.007
Saint Lucia	0.002	0.112	0.001	0.043	0.000	0.024	0.001	0.028	0.000	0.011	0.000	0.005
Saint Vincent and the Grenadines	0.002	0.197	0.001	0.076	0.000	0.047	0.000	0.046	0.000	0.019	0.000	0.009
Suriname	0.005	0.174	0.002	0.056	0.001	0.045	0.001	0.044	0.001	0.018	0.000	0.009

Trinidad and Tobago	0.027	0.112	0.009	0.035	0.006	0.025	0.008	0.031	0.004	0.015	0.001	0.006
Plurinational State of Bolivia	0.101	0.251	0.029	0.072	0.037	0.093	0.014	0.035	0.013	0.032	0.007	0.016
Ecuador	0.155	0.144	0.039	0.036	0.058	0.054	0.023	0.021	0.024	0.022	0.010	0.009
Peru	0.306	0.135	0.093	0.041	0.111	0.049	0.043	0.019	0.039	0.017	0.018	0.008
Colombia	0.435	0.137	0.126	0.039	0.081	0.025	0.144	0.045	0.059	0.018	0.024	0.007
Costa Rica	0.142	0.219	0.040	0.062	0.018	0.028	0.060	0.092	0.017	0.026	0.006	0.010
El Salvador	0.034	0.118	0.008	0.027	0.005	0.016	0.016	0.053	0.004	0.014	0.002	0.006
Guatemala	0.219	0.253	0.048	0.056	0.031	0.036	0.102	0.118	0.023	0.026	0.012	0.014
Honduras	0.062	0.220	0.013	0.046	0.008	0.029	0.030	0.107	0.007	0.024	0.004	0.012
Mexico	1.962	0.149	0.478	0.036	0.155	0.012	1.037	0.079	0.191	0.014	0.082	0.006
Nicaragua	0.034	0.240	0.009	0.061	0.005	0.032	0.015	0.106	0.004	0.025	0.002	0.012
Panama	0.069	0.102	0.021	0.031	0.009	0.013	0.028	0.041	0.007	0.011	0.003	0.005
Brazil	1.777	0.106	0.539	0.032	0.337	0.020	0.651	0.039	0.155	0.009	0.082	0.005
Paraguay	0.074	0.185	0.025	0.063	0.014	0.034	0.025	0.063	0.006	0.014	0.003	0.009
Algeria	0.180	0.097	0.017	0.009	0.059	0.032	0.067	0.036	0.023	0.012	0.011	0.006
Bahrain	0.027	0.066	0.003	0.007	0.010	0.024	0.009	0.022	0.004	0.009	0.001	0.004
Egypt	4.090	0.963	0.369	0.087	0.504	0.119	2.466	0.581	0.497	0.117	0.228	0.054
Islamic Republic of Iran	0.673	0.176	0.054	0.014	0.300	0.078	0.157	0.041	0.112	0.029	0.046	0.012
Iraq	0.375	0.179	0.036	0.017	0.130	0.062	0.135	0.064	0.046	0.022	0.024	0.011
Jordan	0.032	0.068	0.003	0.006	0.011	0.023	0.011	0.024	0.005	0.010	0.002	0.004
Kuwait	0.027	0.018	0.002	0.002	0.009	0.006	0.010	0.007	0.005	0.003	0.001	0.001
Lebanon	0.038	0.163	0.003	0.014	0.017	0.072	0.012	0.051	0.004	0.018	0.002	0.007

Libya	0.130	0.368	0.010	0.028	0.043	0.123	0.051	0.145	0.016	0.046	0.008	0.021
Morocco	0.051	0.036	0.005	0.004	0.018	0.013	0.019	0.014	0.006	0.004	0.003	0.002
Oman	0.047	0.053	0.004	0.005	0.019	0.022	0.014	0.016	0.006	0.007	0.003	0.003
Qatar	0.124	0.069	0.012	0.007	0.050	0.028	0.036	0.020	0.019	0.011	0.006	0.004
Saudi Arabia	0.782	0.089	0.040	0.005	0.309	0.035	0.261	0.030	0.129	0.015	0.041	0.005
Syrian Arab Republic	0.072	0.498	0.006	0.043	0.023	0.161	0.029	0.203	0.009	0.059	0.004	0.028
Turkey	1.231	0.150	0.186	0.023	0.510	0.062	0.316	0.039	0.153	0.019	0.063	0.008
United Arab Emirates	0.401	0.096	0.043	0.010	0.173	0.042	0.108	0.026	0.056	0.013	0.021	0.005
Afghanistan	0.062	0.438	0.004	0.026	0.022	0.154	0.022	0.157	0.006	0.039	0.005	0.036
Bangladesh	0.656	0.157	0.124	0.030	0.197	0.047	0.247	0.059	0.058	0.014	0.018	0.004
Bhutan	0.007	0.239	0.001	0.053	0.002	0.066	0.002	0.082	0.001	0.027	0.000	0.007
India	7.829	0.247	2.104	0.066	2.755	0.087	1.578	0.050	1.078	0.034	0.240	0.008
Nepal	0.124	0.334	0.032	0.087	0.034	0.091	0.040	0.107	0.013	0.035	0.004	0.010
Pakistan	0.867	0.249	0.129	0.037	0.134	0.039	0.498	0.143	0.068	0.019	0.030	0.009
Angola	0.226	0.340	0.037	0.055	0.058	0.087	0.100	0.150	0.016	0.024	0.008	0.012
Central African Republic	0.023	0.896	0.003	0.120	0.006	0.246	0.010	0.409	0.001	0.059	0.001	0.034
Congo	0.052	0.348	0.008	0.053	0.012	0.080	0.024	0.165	0.005	0.031	0.002	0.014
Democratic Republic of the Congo	0.149	0.269	0.017	0.031	0.032	0.058	0.077	0.140	0.012	0.022	0.006	0.012
Equatorial Guinea	0.013	0.108	0.002	0.016	0.003	0.027	0.006	0.047	0.001	0.012	0.001	0.004
Gabon	0.048	0.246	0.009	0.044	0.010	0.053	0.022	0.115	0.005	0.024	0.002	0.008

Burundi	0.009	0.340	0.002	0.060	0.003	0.091	0.003	0.108	0.001	0.041	0.001	0.025
Djibouti	0.008	0.231	0.001	0.039	0.003	0.075	0.002	0.066	0.001	0.031	0.000	0.014
Ethiopia	0.164	0.147	0.025	0.022	0.043	0.038	0.059	0.053	0.019	0.017	0.010	0.009
Kenya	0.226	0.206	0.048	0.044	0.048	0.044	0.071	0.065	0.037	0.034	0.016	0.015
Madagascar	0.033	0.226	0.005	0.036	0.010	0.070	0.009	0.064	0.004	0.030	0.003	0.017
Malawi	0.046	0.375	0.009	0.069	0.014	0.116	0.011	0.093	0.006	0.045	0.003	0.022
Mauritius	0.005	0.047	0.001	0.011	0.002	0.017	0.001	0.012	0.001	0.006	0.000	0.001
Mozambique	0.287	1.775	0.056	0.346	0.111	0.687	0.068	0.419	0.035	0.219	0.015	0.090
Rwanda	0.037	0.331	0.008	0.071	0.009	0.084	0.011	0.101	0.005	0.044	0.002	0.022
Seychelles	0.003	0.200	0.001	0.049	0.001	0.079	0.001	0.044	0.000	0.023	0.000	0.005
Somalia	0.065	0.682	0.009	0.092	0.023	0.243	0.018	0.192	0.007	0.076	0.004	0.044
United Republic of Tanzania	0.193	0.272	0.037	0.053	0.053	0.075	0.053	0.075	0.026	0.037	0.012	0.018
Uganda	0.149	0.367	0.034	0.085	0.042	0.104	0.034	0.083	0.016	0.040	0.009	0.022
Zambia	0.063	0.284	0.012	0.054	0.018	0.080	0.017	0.076	0.008	0.038	0.004	0.017
Botswana	0.044	0.234	0.008	0.045	0.017	0.090	0.011	0.057	0.005	0.029	0.002	0.011
Lesotho	0.031	1.269	0.006	0.239	0.013	0.538	0.007	0.286	0.003	0.145	0.001	0.057
Namibia	0.016	0.129	0.003	0.028	0.006	0.046	0.004	0.032	0.002	0.014	0.001	0.006
South Africa	1.308	0.311	0.294	0.070	0.460	0.109	0.307	0.073	0.185	0.044	0.056	0.013
Kingdom of Eswatini	0.041	0.861	0.008	0.172	0.018	0.387	0.008	0.159	0.005	0.101	0.002	0.036
Zimbabwe	0.219	0.803	0.031	0.115	0.075	0.276	0.070	0.259	0.026	0.094	0.013	0.047
Benin	0.125	0.707	0.019	0.107	0.059	0.332	0.022	0.122	0.015	0.084	0.006	0.034
Burkina Faso	0.263	1.337	0.057	0.287	0.123	0.626	0.044	0.223	0.027	0.138	0.012	0.060
Cameroon	0.342	0.761	0.064	0.143	0.159	0.353	0.052	0.116	0.044	0.097	0.016	0.035

Republic of Cabo Verde	0.017	0.819	0.003	0.147	0.007	0.338	0.004	0.171	0.003	0.125	0.001	0.036
Chad	0.106	0.900	0.018	0.150	0.055	0.464	0.018	0.152	0.010	0.083	0.005	0.042
Cote d'Ivoire	0.121	0.166	0.023	0.031	0.056	0.076	0.017	0.023	0.013	0.017	0.005	0.007
Republic of the Gambia	0.045	2.255	0.008	0.378	0.024	1.174	0.006	0.304	0.005	0.241	0.002	0.103
Ghana	0.334	0.420	0.058	0.073	0.163	0.205	0.053	0.067	0.041	0.052	0.015	0.019
Guinea	0.202	1.182	0.028	0.163	0.098	0.575	0.035	0.206	0.022	0.129	0.010	0.060
Guinea-Bissau	0.022	1.168	0.004	0.191	0.012	0.634	0.003	0.156	0.002	0.113	0.001	0.056
Liberia	0.051	1.451	0.009	0.256	0.026	0.739	0.007	0.203	0.006	0.165	0.002	0.063
Mauritania	0.097	1.047	0.013	0.144	0.048	0.516	0.016	0.177	0.014	0.151	0.004	0.046
Niger	0.097	0.649	0.014	0.094	0.042	0.282	0.020	0.136	0.011	0.074	0.005	0.036
Nigeria	1.037	0.235	0.177	0.040	0.389	0.088	0.234	0.053	0.142	0.032	0.054	0.012
Sao Tome and Principe	0.001	0.121	0.000	0.023	0.000	0.058	0.000	0.018	0.000	0.015	0.000	0.005
Senegal	0.153	0.554	0.021	0.077	0.077	0.278	0.020	0.072	0.024	0.087	0.009	0.032
Togo	0.049	0.589	0.007	0.089	0.022	0.267	0.010	0.117	0.006	0.077	0.003	0.032
American Samoa	0.002	0.235	0.000	0.024	0.001	0.120	0.000	0.048	0.000	0.034	0.000	0.009
Bermuda	0.003	0.039	0.001	0.015	0.001	0.007	0.001	0.010	0.000	0.005	0.000	0.002
Greenland	0.005	0.158	0.002	0.065	0.001	0.018	0.001	0.045	0.001	0.019	0.000	0.011
Guam	0.009	0.145	0.001	0.020	0.005	0.080	0.002	0.025	0.001	0.016	0.000	0.004
Principality of Monaco	0.014	0.164	0.005	0.054	0.002	0.019	0.006	0.069	0.001	0.015	0.001	0.007
Republic of Nauru	0.000	0.143	0.000	0.019	0.000	0.082	0.000	0.022	0.000	0.013	0.000	0.006

Northern Mariana Islands	0.002	0.223	0.000	0.029	0.001	0.117	0.000	0.041	0.000	0.028	0.000	0.007
Republic of Palau	0.001	0.430	0.000	0.066	0.001	0.255	0.000	0.057	0.000	0.040	0.000	0.011
Puerto Rico	0.130	0.122	0.043	0.040	0.028	0.027	0.035	0.033	0.019	0.017	0.005	0.005
Saint Kitts and Nevis	0.002	0.192	0.001	0.064	0.000	0.049	0.000	0.049	0.000	0.021	0.000	0.009
Republic of San Marino	0.002	0.090	0.001	0.034	0.000	0.010	0.001	0.036	0.000	0.007	0.000	0.003
Tuvalu	0.000	0.310	0.000	0.038	0.000	0.158	0.000	0.067	0.000	0.033	0.000	0.012
United States Virgin Islands	0.005	0.104	0.002	0.039	0.001	0.022	0.001	0.025	0.001	0.013	0.000	0.004
Sudan	0.088	0.258	0.010	0.029	0.028	0.082	0.028	0.080	0.011	0.033	0.007	0.021

Supplementary Table 3. VLW and VLW/GDP by nation in 2021 for liver cancer and its pathogenic factors, generated using IE of the VSL at 1.5. All $ values are in 2021 USD, PPP.

IE=1.5	Liver cancer overall		Liver cancer due to alcohol use		Liver cancer due to hepatitis B		Liver cancer due to hepatitis C		Liver cancer due to NASH		Liver cancer due to other causes	
Country	VLW($billion)	VLW/GDP(%)	VLW($billion)	VLW/GDP(%)	VLW($billion)	VLW/GDP(%)	VLW($billion)	VLW/GDP(%)	VLW($billion)	VLW/GDP(%)	VLW($billion)	VLW/GDP(%)
China	15.591	0.087	1.629	0.009	9.020	0.051	3.239	0.018	0.948	0.005	0.727	0.004
Cambodia	0.006	0.017	0.002	0.004	0.002	0.006	0.002	0.005	0.001	0.002	0.000	0.001
Indonesia	0.228	0.019	0.039	0.003	0.064	0.005	0.090	0.008	0.026	0.002	0.007	0.001
Lao People's Democratic Republic	0.003	0.017	0.001	0.004	0.001	0.007	0.001	0.004	0.000	0.001	0.000	0.000
Malaysia	0.125	0.034	0.018	0.005	0.067	0.018	0.023	0.006	0.014	0.004	0.003	0.001
Maldives	0.001	0.020	0.000	0.004	0.000	0.008	0.000	0.005	0.000	0.002	0.000	0.000
Myanmar	0.006	0.009	0.001	0.001	0.002	0.003	0.002	0.003	0.001	0.001	0.000	0.000
Philippines	0.093	0.024	0.024	0.006	0.039	0.010	0.020	0.005	0.008	0.002	0.003	0.001
Sri Lanka	0.006	0.007	0.002	0.002	0.002	0.003	0.002	0.002	0.001	0.001	0.000	0.000
Thailand	0.383	0.076	0.120	0.024	0.150	0.030	0.062	0.012	0.041	0.008	0.009	0.002
Timor-Leste	0.000	0.008	0.000	0.001	0.000	0.003	0.000	0.002	0.000	0.001	0.000	0.000
Socialist Republic of Viet Nam	0.183	0.050	0.052	0.014	0.080	0.022	0.031	0.008	0.015	0.004	0.005	0.001
Fiji	0.001	0.025	0.000	0.003	0.001	0.012	0.000	0.005	0.000	0.003	0.000	0.001
Kiribati	0.000	0.014	0.000	0.001	0.000	0.007	0.000	0.003	0.000	0.002	0.000	0.001
Marshall Islands	0.000	0.022	0.000	0.003	0.000	0.012	0.000	0.004	0.000	0.002	0.000	0.001

Federated States of Micronesia	0.000	0.014	0.000	0.002	0.000	0.008	0.000	0.003	0.000	0.002	0.000	0.001
Papua New Guinea	0.002	0.006	0.000	0.001	0.001	0.003	0.000	0.001	0.000	0.000	0.000	0.000
Samoa	0.000	0.015	0.000	0.002	0.000	0.008	0.000	0.003	0.000	0.002	0.000	0.001
Solomon Islands	0.000	0.008	0.000	0.001	0.000	0.005	0.000	0.001	0.000	0.001	0.000	0.000
Tonga	0.000	0.080	0.000	0.010	0.000	0.042	0.000	0.016	0.000	0.009	0.000	0.003
Vanuatu	0.000	0.011	0.000	0.001	0.000	0.006	0.000	0.002	0.000	0.001	0.000	0.000
Armenia	0.008	0.055	0.002	0.013	0.001	0.010	0.003	0.024	0.001	0.006	0.000	0.002
Azerbaijan	0.027	0.049	0.008	0.014	0.006	0.011	0.010	0.018	0.002	0.004	0.001	0.001
Georgia	0.007	0.035	0.002	0.010	0.001	0.007	0.003	0.013	0.001	0.003	0.000	0.001
Kazakhstan	0.069	0.035	0.019	0.010	0.015	0.008	0.026	0.013	0.007	0.003	0.002	0.001
Kyrgyzstan	0.001	0.007	0.000	0.002	0.000	0.002	0.000	0.003	0.000	0.001	0.000	0.000
Mongolia	0.042	0.274	0.010	0.062	0.010	0.068	0.018	0.116	0.003	0.018	0.001	0.008
Tajikistan	0.001	0.006	0.000	0.001	0.000	0.001	0.000	0.003	0.000	0.000	0.000	0.000
Turkmenistan	0.010	0.020	0.003	0.005	0.002	0.005	0.004	0.008	0.001	0.002	0.000	0.001
Uzbekistan	0.009	0.012	0.002	0.003	0.002	0.003	0.004	0.005	0.001	0.001	0.000	0.000
Albania	0.012	0.067	0.004	0.024	0.003	0.016	0.003	0.018	0.001	0.008	0.000	0.002
Bosnia and Herzegovina	0.018	0.074	0.007	0.028	0.004	0.016	0.005	0.019	0.002	0.009	0.001	0.002
Bulgaria	0.052	0.061	0.024	0.028	0.010	0.012	0.012	0.014	0.005	0.006	0.001	0.002
Croatia	0.040	0.058	0.019	0.028	0.010	0.014	0.005	0.007	0.005	0.007	0.001	0.002
Czech Republic	0.173	0.059	0.087	0.030	0.029	0.010	0.036	0.013	0.016	0.006	0.004	0.001
Hungary	0.061	0.034	0.026	0.014	0.013	0.007	0.014	0.008	0.006	0.004	0.002	0.001

The former Yugoslav Republic of Macedonia	0.013	0.095	0.005	0.038	0.003	0.021	0.003	0.023	0.001	0.010	0.000	0.003
Montenegro	0.004	0.074	0.002	0.029	0.001	0.016	0.001	0.018	0.001	0.009	0.000	0.002
Romania	0.149	0.052	0.066	0.023	0.029	0.010	0.035	0.012	0.015	0.005	0.004	0.001
Serbia	0.063	0.096	0.020	0.030	0.010	0.015	0.026	0.040	0.005	0.008	0.001	0.002
Slovakia	0.086	0.072	0.039	0.032	0.017	0.014	0.020	0.016	0.008	0.007	0.002	0.002
Slovenia	0.062	0.100	0.025	0.040	0.013	0.020	0.016	0.026	0.007	0.011	0.002	0.003
Belarus	0.021	0.030	0.008	0.011	0.004	0.006	0.006	0.009	0.002	0.003	0.001	0.001
Estonia	0.029	0.078	0.012	0.031	0.005	0.013	0.009	0.024	0.003	0.008	0.001	0.002
Latvia	0.025	0.065	0.011	0.028	0.004	0.010	0.007	0.019	0.002	0.006	0.001	0.002
Lithuania	0.047	0.070	0.018	0.028	0.008	0.013	0.014	0.022	0.004	0.007	0.001	0.002
Republic of Moldova	0.005	0.039	0.002	0.015	0.001	0.007	0.002	0.011	0.000	0.003	0.000	0.001
Russian Federation	0.653	0.035	0.242	0.013	0.126	0.007	0.199	0.011	0.064	0.003	0.020	0.001
Ukraine	0.024	0.012	0.008	0.004	0.006	0.003	0.007	0.004	0.002	0.001	0.001	0.000
Brunei Darussalam	0.012	0.088	0.001	0.007	0.006	0.042	0.004	0.029	0.001	0.006	0.000	0.003
Japan	13.373	0.266	1.300	0.026	1.285	0.026	9.830	0.195	0.639	0.013	0.315	0.006
Republic of Korea	4.874	0.268	0.818	0.045	2.638	0.145	0.921	0.051	0.353	0.019	0.143	0.008
Singapore	0.586	0.135	0.042	0.010	0.336	0.077	0.146	0.034	0.032	0.007	0.014	0.003
Australia	1.832	0.118	0.701	0.045	0.214	0.014	0.625	0.040	0.211	0.014	0.077	0.005
New Zealand	0.196	0.077	0.074	0.029	0.026	0.010	0.064	0.025	0.023	0.009	0.009	0.004

Andorra	0.008	0.242	0.002	0.074	0.001	0.027	0.004	0.108	0.001	0.021	0.000	0.011
Austria	0.618	0.129	0.334	0.070	0.034	0.007	0.191	0.040	0.040	0.008	0.018	0.004
Belgium	0.636	0.106	0.220	0.037	0.058	0.010	0.290	0.048	0.045	0.007	0.023	0.004
Cyprus	0.024	0.077	0.008	0.028	0.003	0.009	0.010	0.031	0.002	0.006	0.001	0.003
Denmark	0.372	0.091	0.142	0.035	0.046	0.011	0.143	0.035	0.026	0.006	0.014	0.003
Finland	0.397	0.135	0.134	0.046	0.047	0.016	0.169	0.057	0.031	0.011	0.015	0.005
France	4.281	0.144	1.245	0.042	0.467	0.016	2.066	0.070	0.333	0.011	0.164	0.006
Germany	5.548	0.128	2.382	0.055	0.499	0.011	1.945	0.045	0.455	0.010	0.264	0.006
Greece	0.173	0.079	0.049	0.022	0.059	0.027	0.044	0.020	0.014	0.007	0.007	0.003
Iceland	0.025	0.096	0.009	0.034	0.003	0.013	0.010	0.037	0.002	0.008	0.001	0.004
Ireland	0.533	0.100	0.190	0.036	0.059	0.011	0.216	0.041	0.045	0.008	0.022	0.004
Israel	0.198	0.040	0.050	0.010	0.027	0.006	0.092	0.019	0.018	0.004	0.010	0.002
Italy	2.549	0.117	0.604	0.028	0.231	0.011	1.533	0.070	0.126	0.006	0.055	0.003
Luxembourg	0.124	0.144	0.046	0.053	0.014	0.016	0.050	0.059	0.009	0.011	0.005	0.006
Malta	0.011	0.057	0.004	0.021	0.001	0.007	0.004	0.022	0.001	0.004	0.000	0.002
Netherlands	0.802	0.076	0.330	0.031	0.104	0.010	0.253	0.024	0.077	0.007	0.037	0.004
Norway	0.560	0.111	0.174	0.035	0.067	0.013	0.249	0.050	0.043	0.008	0.026	0.005
Portugal	0.258	0.101	0.103	0.040	0.030	0.012	0.097	0.038	0.018	0.007	0.009	0.004
Spain	1.580	0.108	0.447	0.031	0.127	0.009	0.854	0.058	0.097	0.007	0.054	0.004
Sweden	0.526	0.083	0.215	0.034	0.036	0.006	0.202	0.032	0.046	0.007	0.026	0.004
Switzerland	1.124	0.138	0.416	0.051	0.142	0.017	0.445	0.055	0.079	0.010	0.041	0.005
United Kingdom of Great Britain and Northern Ireland	4.333	0.138	1.491	0.047	0.518	0.016	1.786	0.057	0.358	0.011	0.174	0.006

Argentina	0.047	0.010	0.013	0.003	0.009	0.002	0.017	0.004	0.005	0.001	0.003	0.001
Chile	0.099	0.031	0.029	0.009	0.016	0.005	0.038	0.012	0.011	0.004	0.005	0.002
Uruguay	0.019	0.031	0.006	0.009	0.003	0.006	0.007	0.011	0.002	0.003	0.001	0.001
Canada	2.127	0.106	1.054	0.052	0.130	0.006	0.504	0.025	0.337	0.017	0.098	0.005
United States of America	28.402	0.120	8.371	0.035	3.051	0.013	11.331	0.048	3.220	0.014	2.355	0.010
Antigua and Barbuda	0.000	0.025	0.000	0.009	0.000	0.006	0.000	0.006	0.000	0.002	0.000	0.001
Commonwealth of the Bahamas	0.004	0.036	0.001	0.012	0.001	0.010	0.001	0.008	0.000	0.004	0.000	0.002
Barbados	0.002	0.043	0.001	0.015	0.000	0.008	0.001	0.012	0.000	0.005	0.000	0.002
Belize	0.000	0.011	0.000	0.003	0.000	0.003	0.000	0.003	0.000	0.001	0.000	0.001
Dominica	0.000	0.033	0.000	0.011	0.000	0.007	0.000	0.009	0.000	0.004	0.000	0.001
Dominican Republic	0.010	0.011	0.003	0.003	0.003	0.003	0.003	0.003	0.001	0.001	0.001	0.001
Grenada	0.000	0.023	0.000	0.009	0.000	0.005	0.000	0.006	0.000	0.002	0.000	0.001
Guyana	0.001	0.015	0.000	0.005	0.000	0.004	0.000	0.004	0.000	0.002	0.000	0.001
Haiti	0.001	0.003	0.000	0.001	0.000	0.001	0.000	0.001	0.000	0.000	0.000	0.000
Jamaica	0.002	0.011	0.000	0.003	0.000	0.002	0.000	0.003	0.000	0.001	0.000	0.001
Saint Lucia	0.000	0.018	0.000	0.007	0.000	0.004	0.000	0.004	0.000	0.002	0.000	0.001
Saint Vincent and the Grenadines	0.000	0.026	0.000	0.010	0.000	0.006	0.000	0.006	0.000	0.003	0.000	0.001
Suriname	0.000	0.014	0.000	0.005	0.000	0.004	0.000	0.004	0.000	0.001	0.000	0.001
Trinidad and Tobago	0.007	0.030	0.002	0.010	0.002	0.007	0.002	0.008	0.001	0.004	0.000	0.002

Plurinational State of Bolivia	0.006	0.014	0.002	0.004	0.002	0.005	0.001	0.002	0.001	0.002	0.000	0.001
Ecuador	0.015	0.014	0.004	0.003	0.006	0.005	0.002	0.002	0.002	0.002	0.001	0.001
Peru	0.033	0.015	0.010	0.004	0.012	0.005	0.005	0.002	0.004	0.002	0.002	0.001
Colombia	0.043	0.013	0.012	0.004	0.008	0.003	0.014	0.004	0.006	0.002	0.002	0.001
Costa Rica	0.028	0.043	0.008	0.012	0.004	0.006	0.012	0.018	0.003	0.005	0.001	0.002
El Salvador	0.003	0.009	0.001	0.002	0.000	0.001	0.001	0.004	0.000	0.001	0.000	0.000
Guatemala	0.017	0.020	0.004	0.004	0.002	0.003	0.008	0.009	0.002	0.002	0.001	0.001
Honduras	0.003	0.010	0.001	0.002	0.000	0.001	0.001	0.005	0.000	0.001	0.000	0.001
Mexico	0.312	0.024	0.076	0.006	0.025	0.002	0.165	0.013	0.030	0.002	0.013	0.001
Nicaragua	0.001	0.009	0.000	0.002	0.000	0.001	0.001	0.004	0.000	0.001	0.000	0.000
Panama	0.016	0.024	0.005	0.007	0.002	0.003	0.006	0.010	0.002	0.003	0.001	0.001
Brazil	0.222	0.013	0.067	0.004	0.042	0.003	0.081	0.005	0.019	0.001	0.010	0.001
Paraguay	0.007	0.018	0.002	0.006	0.001	0.003	0.002	0.006	0.001	0.001	0.000	0.001
Algeria	0.012	0.006	0.001	0.001	0.004	0.002	0.005	0.002	0.002	0.001	0.001	0.000
Bahrain	0.011	0.026	0.001	0.003	0.004	0.009	0.004	0.009	0.001	0.004	0.001	0.001
Egypt	0.254	0.060	0.023	0.005	0.031	0.007	0.153	0.036	0.031	0.007	0.014	0.003
Islamic Republic of Iran	0.047	0.012	0.004	0.001	0.021	0.005	0.011	0.003	0.008	0.002	0.003	0.001
Iraq	0.029	0.014	0.003	0.001	0.010	0.005	0.011	0.005	0.004	0.002	0.002	0.001
Jordan	0.002	0.005	0.000	0.000	0.001	0.002	0.001	0.002	0.000	0.001	0.000	0.000
Kuwait	0.014	0.009	0.001	0.001	0.004	0.003	0.005	0.003	0.002	0.002	0.001	0.000
Lebanon	0.002	0.011	0.000	0.001	0.001	0.005	0.001	0.003	0.000	0.001	0.000	0.000
Libya	0.010	0.029	0.001	0.002	0.003	0.010	0.004	0.012	0.001	0.004	0.001	0.002
Morocco	0.003	0.002	0.000	0.000	0.001	0.001	0.001	0.001	0.000	0.000	0.000	0.000

Oman	0.014	0.015	0.001	0.001	0.006	0.006	0.004	0.005	0.002	0.002	0.001	0.001
Qatar	0.125	0.070	0.012	0.007	0.050	0.028	0.037	0.020	0.019	0.011	0.006	0.004
Saudi Arabia	0.326	0.037	0.017	0.002	0.129	0.015	0.109	0.012	0.054	0.006	0.017	0.002
Syrian Arab Republic	0.001	0.006	0.000	0.001	0.000	0.002	0.000	0.002	0.000	0.001	0.000	0.000
Turkey	0.186	0.023	0.028	0.003	0.077	0.009	0.048	0.006	0.023	0.003	0.009	0.001
United Arab Emirates	0.250	0.060	0.027	0.006	0.108	0.026	0.067	0.016	0.035	0.008	0.013	0.003
Afghanistan	0.000	0.003	0.000	0.000	0.000	0.001	0.000	0.001	0.000	0.000	0.000	0.000
Bangladesh	0.027	0.006	0.005	0.001	0.008	0.002	0.010	0.002	0.002	0.001	0.001	0.000
Bhutan	0.000	0.014	0.000	0.003	0.000	0.004	0.000	0.005	0.000	0.002	0.000	0.000
India	0.292	0.009	0.079	0.002	0.103	0.003	0.059	0.002	0.040	0.001	0.009	0.000
Nepal	0.003	0.007	0.001	0.002	0.001	0.002	0.001	0.002	0.000	0.001	0.000	0.000
Pakistan	0.021	0.006	0.003	0.001	0.003	0.001	0.012	0.004	0.002	0.000	0.001	0.000
Angola	0.007	0.011	0.001	0.002	0.002	0.003	0.003	0.005	0.001	0.001	0.000	0.000
Central African Republic	0.000	0.008	0.000	0.001	0.000	0.002	0.000	0.004	0.000	0.001	0.000	0.000
Congo	0.002	0.015	0.000	0.002	0.000	0.003	0.001	0.007	0.000	0.001	0.000	0.001
Democratic Republic of the Congo	0.001	0.003	0.000	0.000	0.000	0.001	0.001	0.001	0.000	0.000	0.000	0.000
Equatorial Guinea	0.001	0.012	0.000	0.002	0.000	0.003	0.001	0.005	0.000	0.001	0.000	0.000
Gabon	0.006	0.031	0.001	0.006	0.001	0.007	0.003	0.015	0.001	0.003	0.000	0.001
Burundi	0.000	0.001	0.000	0.000	0.000	0.000	0.000	0.000	0.000	0.000	0.000	0.000
Djibouti	0.000	0.011	0.000	0.002	0.000	0.004	0.000	0.003	0.000	0.002	0.000	0.001

Ethiopia	0.003	0.002	0.000	0.000	0.001	0.001	0.001	0.001	0.000	0.000	0.000	0.000
Kenya	0.008	0.007	0.002	0.002	0.002	0.002	0.002	0.002	0.001	0.001	0.001	0.001
Madagascar	0.000	0.002	0.000	0.000	0.000	0.001	0.000	0.001	0.000	0.000	0.000	0.000
Malawi	0.001	0.004	0.000	0.001	0.000	0.001	0.000	0.001	0.000	0.000	0.000	0.000
Mauritius	0.001	0.007	0.000	0.001	0.000	0.002	0.000	0.002	0.000	0.001	0.000	0.000
Mozambique	0.003	0.016	0.001	0.003	0.001	0.006	0.001	0.004	0.000	0.002	0.000	0.001
Rwanda	0.001	0.005	0.000	0.001	0.000	0.001	0.000	0.001	0.000	0.001	0.000	0.000
Seychelles	0.001	0.045	0.000	0.011	0.000	0.018	0.000	0.010	0.000	0.005	0.000	0.001
Somalia	0.001	0.007	0.000	0.001	0.000	0.002	0.000	0.002	0.000	0.001	0.000	0.000
United Republic of Tanzania	0.004	0.005	0.001	0.001	0.001	0.001	0.001	0.001	0.001	0.001	0.000	0.000
Uganda	0.002	0.006	0.001	0.001	0.001	0.002	0.001	0.001	0.000	0.001	0.000	0.000
Zambia	0.001	0.006	0.000	0.001	0.000	0.002	0.000	0.001	0.000	0.001	0.000	0.000
Botswana	0.005	0.029	0.001	0.005	0.002	0.011	0.001	0.007	0.001	0.004	0.000	0.001
Lesotho	0.001	0.023	0.000	0.004	0.000	0.010	0.000	0.005	0.000	0.003	0.000	0.001
Namibia	0.001	0.009	0.000	0.002	0.000	0.003	0.000	0.002	0.000	0.001	0.000	0.000
South Africa	0.141	0.034	0.032	0.008	0.050	0.012	0.033	0.008	0.020	0.005	0.006	0.001
Kingdom of Eswatini	0.003	0.055	0.001	0.011	0.001	0.025	0.000	0.010	0.000	0.006	0.000	0.002
Zimbabwe	0.006	0.023	0.001	0.003	0.002	0.008	0.002	0.008	0.001	0.003	0.000	0.001
Benin	0.003	0.016	0.000	0.002	0.001	0.007	0.000	0.003	0.000	0.002	0.000	0.001
Burkina Faso	0.004	0.021	0.001	0.004	0.002	0.010	0.001	0.003	0.000	0.002	0.000	0.001
Cameroon	0.010	0.022	0.002	0.004	0.005	0.010	0.001	0.003	0.001	0.003	0.000	0.001
Republic of Cabo Verde	0.001	0.053	0.000	0.009	0.000	0.022	0.000	0.011	0.000	0.008	0.000	0.002

Chad	0.001	0.011	0.000	0.002	0.001	0.005	0.000	0.002	0.000	0.001	0.000	0.000
Cote d'Ivoire	0.005	0.007	0.001	0.001	0.002	0.003	0.001	0.001	0.001	0.001	0.000	0.000
Republic of the Gambia	0.001	0.031	0.000	0.005	0.000	0.016	0.000	0.004	0.000	0.003	0.000	0.001
Ghana	0.014	0.017	0.002	0.003	0.007	0.008	0.002	0.003	0.002	0.002	0.001	0.001
Guinea	0.004	0.025	0.001	0.003	0.002	0.012	0.001	0.004	0.000	0.003	0.000	0.001
Guinea-Bissau	0.000	0.018	0.000	0.003	0.000	0.010	0.000	0.002	0.000	0.002	0.000	0.001
Liberia	0.001	0.017	0.000	0.003	0.000	0.009	0.000	0.002	0.000	0.002	0.000	0.001
Mauritania	0.003	0.034	0.000	0.005	0.002	0.017	0.001	0.006	0.000	0.005	0.000	0.001
Niger	0.001	0.007	0.000	0.001	0.000	0.003	0.000	0.001	0.000	0.001	0.000	0.000
Nigeria	0.035	0.008	0.006	0.001	0.013	0.003	0.008	0.002	0.005	0.001	0.002	0.000
Sao Tome and Principe	0.000	0.005	0.000	0.001	0.000	0.002	0.000	0.001	0.000	0.001	0.000	0.000
Senegal	0.004	0.015	0.001	0.002	0.002	0.008	0.001	0.002	0.001	0.002	0.000	0.001
Togo	0.001	0.010	0.000	0.001	0.000	0.004	0.000	0.002	0.000	0.001	0.000	0.001
American Samoa	0.000	0.054	0.000	0.005	0.000	0.028	0.000	0.011	0.000	0.008	0.000	0.002
Bermuda	0.004	0.061	0.002	0.023	0.001	0.011	0.001	0.016	0.001	0.008	0.000	0.002
Greenland	0.004	0.128	0.002	0.052	0.000	0.014	0.001	0.037	0.000	0.015	0.000	0.009
Guam	0.005	0.080	0.001	0.011	0.003	0.044	0.001	0.014	0.001	0.009	0.000	0.002
Principality of Monaco	0.042	0.486	0.014	0.160	0.005	0.057	0.018	0.204	0.004	0.045	0.002	0.019
Republic of Nauru	0.000	0.032	0.000	0.004	0.000	0.019	0.000	0.005	0.000	0.003	0.000	0.001
Northern Mariana Islands	0.001	0.065	0.000	0.008	0.000	0.034	0.000	0.012	0.000	0.008	0.000	0.002

Republic of Palau	0.000	0.087	0.000	0.013	0.000	0.052	0.000	0.012	0.000	0.008	0.000	0.002
Puerto Rico	0.062	0.058	0.020	0.019	0.013	0.013	0.017	0.016	0.009	0.008	0.003	0.002
Saint Kitts and Nevis	0.000	0.053	0.000	0.018	0.000	0.014	0.000	0.014	0.000	0.006	0.000	0.002
Republic of San Marino	0.001	0.069	0.000	0.026	0.000	0.007	0.001	0.028	0.000	0.005	0.000	0.003
Tuvalu	0.000	0.029	0.000	0.004	0.000	0.015	0.000	0.006	0.000	0.003	0.000	0.001
United States Virgin Islands	0.003	0.064	0.001	0.024	0.001	0.014	0.001	0.015	0.000	0.008	0.000	0.002
Sudan	0.001	0.003	0.000	0.000	0.000	0.001	0.000	0.001	0.000	0.000	0.000	0.000
